# Circular RNAs in EMT-driven metastasis regulation: modulation of cancer cell plasticity, tumorigenesis and therapy resistance

**DOI:** 10.1007/s00018-024-05236-w

**Published:** 2024-05-11

**Authors:** Milad Ashrafizadeh, Jingyuan Dai, Pedram Torabian, Noushin Nabavi, Amir Reza Aref, Alaa A. A. Aljabali, Murtaza Tambuwala, Minglin Zhu

**Affiliations:** 1 Department of Radiation Oncology, Shandong Provincial Key Laboratory of Radiation Oncology, Shandong Cancer Hospital and Institute, Shandong First Medical University, Shandong Academy of Medical Sciences, Jinan, Shandong 250000 China; 2 Department of General Surgery and Integrated Chinese and Western Medicine, Institute of Precision Diagnosis and Treatment of Gastrointestinal Tumors, Carson International Cancer Center, Shenzhen University General Hospital, Shenzhen University, Shenzhen, Guangdong 518060 China; 3 Shanghai Institute of Cardiovascular Diseases, Zhongshan Hospital, Fudan University, Shanghai, 200032 China; 4 School of computer science and information systems, Northwest Missouri State University, Maryville, MO 64468 USA; 5 Cumming School of Medicine, Arnie Charbonneau Cancer Research Institute, University of Calgary, Calgary, AB T2N 4Z6 Canada; 6 Department of Medical Sciences, University of Calgary, Calgary, AB T2N 4Z6 Canada; 7 Department of Urologic Sciences and Vancouver Prostate Centre, University of British Columbia, Vancouver, BC V6H3Z6 Canada; 8 Belfer Center for Applied Cancer Science, Dana-Farber Cancer Institute, Harvard Medical School, Boston, MA USA; 9 Department of Translational Sciences, Xsphera Biosciences Inc. Boston, Boston, MA USA; 10 Faculty of Pharmacy, Department of Pharmaceutics and Pharmaceutical Technology, Yarmouk University, Irbid, Jordan; 11 Lincoln Medical School, University of Lincoln, Brayford Pool Campus, Lincoln, LN6 7TS UK; 12 Department of Cardiovascular Surgery, Zhongnan Hospital of Wuhan University, Wuhan, Hubei China; 13 Hubei Provincial Engineering Research Center of Minimally Invasive Cardiovascular Surgery, Wuhan, Hubei 430071 China; 14 College of Pharmacy, Ras Al Khaimah Medical and Health Sciences University, Ras Al Khaimah, United Arab Emirates

**Keywords:** RNA transcripts, circRNAs, cancer malignancy, EMT, cancer cell plasticity, Drug resistance

## Abstract

The non-coding RNAs comprise a large part of human genome lack of capacity in encoding functional proteins. Among various members of non-coding RNAs, the circular RNAs (circRNAs) have been of importance in the pathogenesis of human diseases, especially cancer. The circRNAs have a unique closed loop structure and due to their stability, they are potential diagnostic and prognostic factors in cancer. The increasing evidences have highlighted the role of circRNAs in the modulation of proliferation and metastasis of cancer cells. On the other hand, metastasis has been responsible for up to 90% of cancer-related deaths in patients, requiring more investigation regarding the underlying mechanisms modulating this mechanism. EMT enhances metastasis and invasion of tumor cells, and can trigger resistance to therapy. The cells demonstrate dynamic changes during EMT including transformation from epithelial phenotype into mesenchymal phenotype and increase in N-cadherin and vimentin levels. The process of EMT is reversible and its reprogramming can disrupt the progression of tumor cells. The aim of current review is to understanding the interaction of circRNAs and EMT in human cancers and such interaction is beyond the regulation of cancer metastasis and can affect the response of tumor cells to chemotherapy and radiotherapy. The onco-suppressor circRNAs inhibit EMT, while the tumor-promoting circRNAs mediate EMT for acceleration of carcinogenesis. Moreover, the EMT-inducing transcription factors can be controlled by circRNAs in different human tumors.

## Introduction

**Fig. 1:**
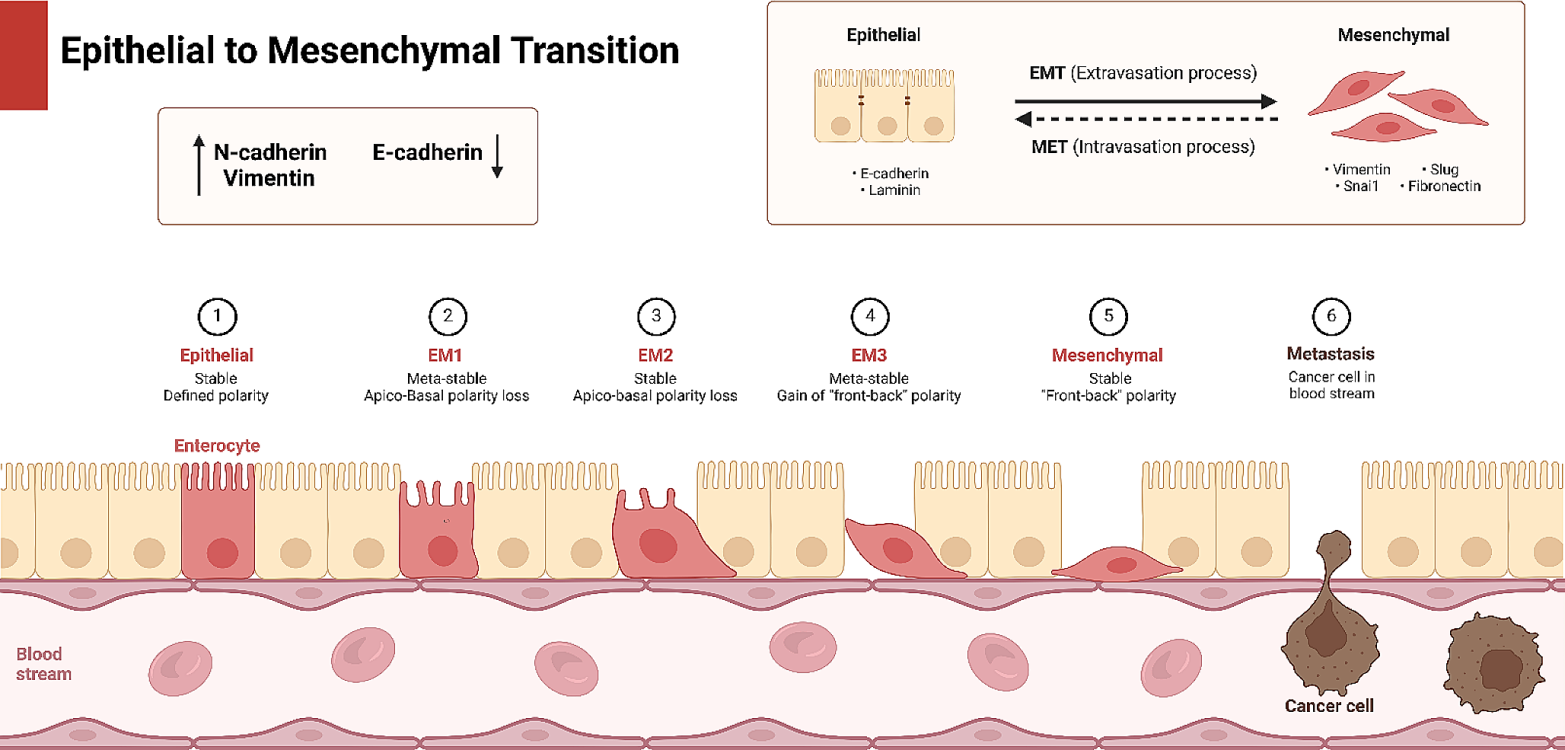
A summary of EMT mechanism [5]. The EMT includes morphological and biochemical alterations that can occur simultaneously. The biochemical features include the downregulation of E-cadherin and upregulation of vimentin and N-cadherin that can participate in EMT induction. The epithelial cells are changed into mesenchymal cells during EMT that demonstrate high levels of Slug, Snail1, vimentin and fibronectin. The occurrence of EMT can result in escape of a number of cells from other population of colony that diffuse into blood steam to reach to a new site for the establishment of a new colony, participating in the metastasis and progression of tumor

Since cancer mortality is associated with metastasis, it is essential to emphasize the role of factors that regulate tumor invasion [1]. Therefore, improving treatment and the survival of patients rely on reducing invasion and the malignancy of tumors. Metastasis is one of the hallmarks of tumor and was coined in 1829 by Jean Claude [2]. The word “metastasis” has Greek roots and means “displacement”, with “meta” meaning next and “statis” meaning placement [3]. Metastasis refers to the process in which tumor cells migrate to another part of the body from their primary site to form new cancer cells, leading to death [4]. Cancer metastasis can be modulated via various mechanisms, such as EMT. Increasing evidence reveals that EMT mechanisms have a potential role in tumor invasion and drug insensitivity (Fig. 1).

**Fig. 2:**
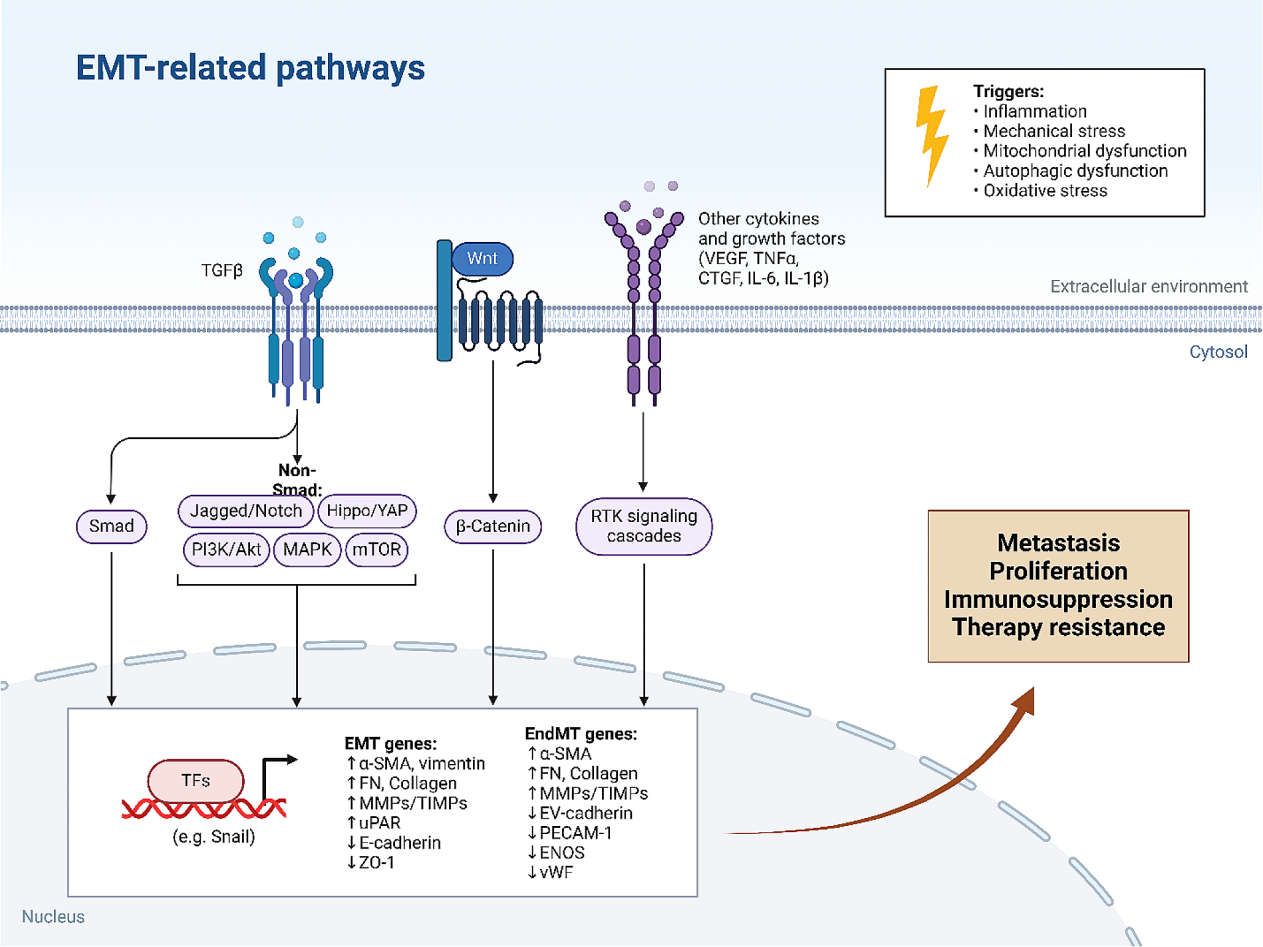
An overview of a number of pathways regulating EMT. TGF-β, Wnt, PI3K/Akt, mTOR, YAP, MAPK and RTK are a number of molecular interactions contributing to the regulation of EMT. Such pathways are related to the proteins that finally translocate into the nucleus to regulate expression levels of EMT-related genes. The process of EMT is vital for carcinogenesis, since EMT participates in metastasis, therapy resistance and immunosuppression in human cancers. (Created by Biorender.com)

**Fig. 3:**
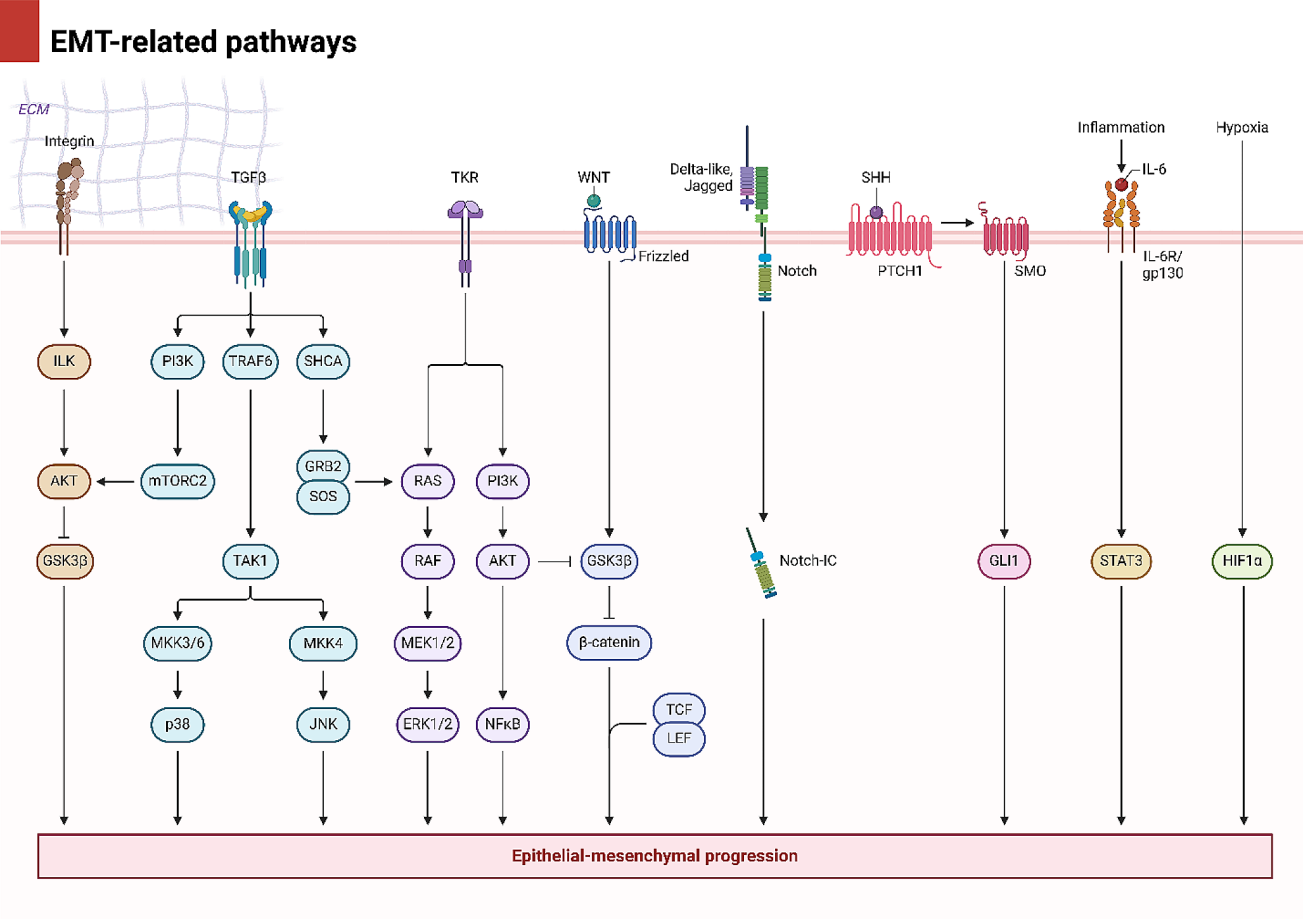
The other mechanisms regulating EMT [20]. A number of these pathways demonstrate interaction in the regulation of EMT such as TGF-β that modulates mTORC2 and the integrin that regulates AKT. Finally, mTOCR2 can induce AKT. TKB, Wnt/β-catenin, Notch, GLI1, STAT3 and HIF-1α are among the other pathways regulating EMT. (Created by Biorender.com)

EMT causes a loss of cell-cell and cell-extracellular matrix adhesion, causing cells with a mesenchymal phenotype to detach from their primary site and separate from surrounding tissues. The process of EMT is not only vital for cancer metastasis but is essential for embryogenesis, tissue fibrosis, and wound healing [6]. Upon EMT induction, the motility of cancer cells increases, and epithelial cells lose their apical-basal polarity in order to transform into mesenchymal cells [7]. The generation of adherens junctions by E-cadherin is vital for cell adhesion and polarity in epithelia and leads to cell-cell attachment and recruitment of signaling complexes [8, 9]. The earliest step in the process of EMT is the loss of E-cadherin levels. EMT-TFs mediate epithelial reorganization via changing and decreasing E-cadherin levels [10, 11]. EMT is associated with epithelial-mesenchymal transitions, changes in EMT-TF expression levels, including ZEB proteins, TGF-β, Twist and Snail, as well as Wnt/β-catenin and Notch regulation. The induction of EMT and overexpression of PD-L1 by ERK signaling can cause drug resistance lung tumor. Suppressing PAR2 leads to inhibition of ERK-induced EMT to inhibit osimertinib resistance in lung cancer [12]. Also, Bakuchiol administration is critical for suppressing EMT and cancer invasion by downregulating TGF-β [13]. Low expression levels of ADRB2 by cardamonin are advantageous in inhibiting EMT and reducing the metastasis of colorectal tumor [14]. Besides, activation of Hedgehog signaling is vital for EMT induction in breast tumor [15]. Hence, the regulation of EMT in cancer occurs via various molecular pathways, and targeting inducers of EMT is therefore beneficial in reversing tumor invasion [16–18]. Interestingly, non-coding RNAs can control EMT mechanism [19], and the purpose of the present review is to shed some light on the function of circular RNAs (circRNAs) in regulating EMT and tumor invasion. The Figs. 2 and 3 demonstrate an overview of EMT-related pathways.

## The function of EMT in tumorigenesis

Despite the valuable functions of EMT during the physiological and developmental stages, the function of EMT in cancer is oncogenic. Although EMT has been confirmed as a modulator of cancer migration, the increasing evidences have shown that EMT can also mediate the drug resistance [21, 22]. Therefore, the recent experiments have emphasized on highlighting the underlying mechanisms modulating EMT in human cancers. The androgen receptor has shown ability in increasing prostate cancer invasion. The androgen receptor stimulates eIF5A2 expression to enhance vimentin and N-cadherin levels for EMT induction in prostate tumor [23]. In pancreatic cancer, the induction of EMT can significantly increase cancer metastasis and mediate poor prognosis. The upregulation of MACC1 in nucleus and itsinteraction with SNAI1 as EMT regulator can significantly enhance the metastasis of pancreatic tumor [24]. The lncRNA NRON has been shown as an inducer of metastasis and progression in bladder tumor. LncRNA NRON can upregulate EZH2 to stimulate EMT for bladder tumor invasion [25]. Noteworthy, the induction of EMT can also participate in the immunosuppression in human cancers. The ZEB1 is an inducer of EMT in which can stimulate exocytotoic vesicular trafficking through release of Rab6A and Rab8A. Then, MMP14-mediated focal adhesion turnover occurs in lung cancer and induces CD8 + T cell exhaustion [26]. In cholangiocarcinoma, the upregulation of PLCB1 occurs to induce PI3K/Akt axis. Then, phosphorylation of GSK-3β pccurs to increase Snail levels for induction of EMT and increasing cancer progression. However, miR-26b-5p suppresses EMT through PLCB1 downregulation [27]. These experiments provide the insight that EMT has a versatile function in tumorigenesis and it is not limited to metastasis, while it can affect therapy response and impair the immune system.

## CircRNAs: an overview in oncology

More than four decades ago, a new kind of RNA molecule without the capability of protein coding was recognized as circular RNA (circRNA). The first circRNA was identified by Sanger and colleagues with a covalently closed loop structure in plant viroids [28]. After that, circRNAs were recognized in eukaryotic cells and viruses [29, 30]. In 1991, the first mammalian circular transcript from the DCC gene was identified in cancerous and non-cancerous cells. Nevertheless, circRNAs were not easily accepted after their discovery and were initially dismissed. They were believed to be by-products of erroneous splicing and transcription errors, and their function was called into question [31]. Progress and efforts on the identification of these RNA molecules were stopped until recently with the discovery of high-throughput sequencing technology and bioinformatic tools. A high number of circRNAs, 32,000 human exonic circRNAs, have been discovered to date [32]. As stable non-coding RNA molecules, circRNAs are mainly found in the cytoplasm of eukaryotic cells, although a number of them, such as intronic circRNAs, can be present in the nucleus [33]. Linear RNA molecules, such as lncRNAs and miRNAs, have a 5′ end cap and a 3′ end poly(A) tail, but circRNAs have a unique structure that lacks both 5′ and 3′ ends. Therefore, expression of circRNAs is stable, and their stability is high due to a lack of degradation by endonucleases [34]. CircRNAs are able to sponge miRNAs in the cytoplasm and accelerate the procedure of protein translation. Moreover, circRNAs regulate the transcription process by interacting with RBPs [35–37]. The action of circRNAs in affecting tumor progression is mainly performed via sponging miRNAs. For example, circCCDC85A sponges miR-550a-5p, increasing MOB1A levels and impairing breast tumor progression [38]. Tumor progression and proliferation are tightly regulated by the function of circRNAs [39]. Hsa-circ-0001013 upregulates TWSG1 via miR-136 inhibition, promoting gastric tumorigenesis [40]. The circMET contributes to tamoxifen resistance in breast tumor via increasing AHR level [41]. Furthermore, stabilization of ATP7A and upregulation of PHLPP2 by circPBX3 and circ-0001017, respectively, can lead to tumorigenesis [42, 43].

There are three subcategories of circRNAs based on the distribution and biogenesis [44]. The first subclass is exonic circRNAs (ecRNAs) that are generated through back-splicing of the 5′ splice site (splice donor site) to a 3’ splice site (splice acceptor site) [45, 46]. The second subclass is intronic circRNAs (ciRNAs) that are originated from the intronic lariat precursoes escaping from the debranching step of canonical linear splicing [47]. The third subclass is exon-intron circRNA (ElciRNAs) that increase the expression of parental genes in *cis* through circularizing with the retained intronic sequences among circularized exosones [48]. Moreover, a new kind of circRNAs have been recognized known as mitochondria-encoded circRNAs (mecciRNA) that their biogenesis occurs through a splicing-idependent pathway and can function as molecular chaperons to accelerate the mitochondrial entry of nuclear-encoded proteins [49]. The biogenesis of circRNAs has been highlighted somehow and there are still many unknown aspects. The circRNAs have been shown to be derived from pre-mRNAs and they undergo canonical spliceosomal machinery [50]. The back-splicing reactions in circRNAs require the covalent linking of the 3′ splice site with the 5’ splice site, occurring during biogenesis of circRNAs and can be accelerated through reverse complementary Alu repeats flanking the circularized exon [51].

The function of circRNAs occurs through four distinct mechanisms [52]. For influencing the expression of genes, the circRNAs affect the parental gene mRNA mediated through interaction with RNA binding proteins [53–55]. Notably, the function of circRNAs is also related to acting as ceRNAs to spong miRNAs [56–58]. Moreover, the circRNAs have been shown to regulate the immune responses [59–61]. Although circRNAs are mainly believed as non-coding RNA factors, a variety of circRNAs have been shown to exert their function through encoding proteins [62–64]. The circRNAs are considered as potential factors in carcinogenesis. The circ-403,658 increases LDHA expression to induce glycolysis in bladder tumor [65]. Moreover, circ-103,809 facilitates the tumorigenesis of hepatocellular carcinoma through acting as tumor-promoting factor [66]. Table 1 summarizes the function of circRNAs in tumors. Table 2 summarizes the role of circRNAs in the controlling EMT.

**Table 1:** A summary of circRNAs function

CircRNA	Cancer type	Remark	Reference
Hsa_circ_0136666	Gastric cancer	Sponging miR-375-3p to increase PRKDC expression and enhance PD-L1 levels in immune evasion	[67]
Hsa_circ_0006401	Colorectal cancer	Downregulation of circRNA decreases growth and invasion of tumor cells	[68]
Circ_0004140	Lung adenocarcinoma	Circ_0004140 sponges miR-1184 to upregulate CCL22 in tumorigenesis	[69]
Hsa_circ_0000437	Gastric cancer	Increasing lymph node metastasis through HSPA2/ERK axis regulation	[70]
Hsa_circ_0003258	Prostate cancer	Sponging miR-653-5p and complexation with IGF2BP3 to enhance invasion	[71]
Circ_0000235	Bladder cancer	Downregulation of miR-330-5p to stimulate glycolysis	[72]
Circ_0042881	Breast cancer	EIF4A3 increases circ-0042881 levels to stimulate RAS pathway in tumorigenesis	[73]
Hsa_circ_0060467	Breast cancer	Hsa_circ_0060467 inhibits miR-1205 and mediates complexation with eIF4A3 to enhance liver invasion	[74]
Circ-hnRNPU	Gastric cancer	Suppression of NONO-mediated c-Myc transactivation and mRNA stabilization vital for glycolysation and tumorigenesis	[75]

**Table 2:** The circRNAs-associated EMT control

CircRNA	Effect on EMT	Cancer type	Remarks	References
Hsa_circ_0094606	Induction	Prostate cancer	Hsa_circ_0094606 attaches to PRMT1 to mediate M2 polarization of macrophages EMT induction	[76]
Hsa_circ_0013561	Induction	Ovarian cancer	Hsa_circ_0013561 upregulates ANXA2 through miR-23b-3p suppression in EMT induction	[77]
Circ_0087429	Suppression	Cervical cancer	EIF4A3 inhibits circ-0087429 to mediate EMT	[78]
Hsa_circ_0001666	Suppression	Colorectal cancer	Hsa_circ_0001666 increases PCDH10 level through miR-576-5p disruption to inhibit EMT	[79]
Hsa_circ_0006732	Induction	Colorectal cancer	Hsa_circ_0006732 downregulates miR-127-3p to increase Rab3D levels in EMT induction	[80]
Circ_0000799	Induction	Colorectal cancer	Sponging miR-647	[81]
Hsa_circ_0000497 and hsa_circ_0000918	Induction	Ovarian cancer	Hsa_circ_0000497 and hsa_circ_0000918 regulate miRNAs to induce EMT in ascites	[82]
Circ_0001589	Induction	Cervical cancer	Circ_0001589 sponges miR-1248 to upregulate HMGB1 in EMT induction	[83]
Circ-OXCT1	Suppression	Gastric cancer	Circ-OXCT1 increases SMAD4 expression to promote N-cadherin and vimentin levels and reduce E-cadherin levels	[84]
Circ_0003221	Induction	Cervical cancer	Circ_0003221 increases S100A14 level by miR-139-3p inhibition	[85]
CircRNA circ_0001666	Induction	Pancreatic cancer	Downregulation of circRNA circ_0001666 increasing miR-1251 level, while it reduces SOX4 level to impair EMT	[86]
Circ_0058106	Induction	Hypopharyngeal squamous cell carcinoma	miR-185-3p downregulation to induce Wnt2b/β-catenin/c-Myc axis	[87]
Circ-STK39	Induction	Pancreatic cancer	Circ-STK39 inhibits miR-140-3p to increase TRAM2 levels	[88]
Hsa_circ_0009092	Suppression	Colorectal cancer	Hsa_circ_0009092 sponges miR-665 to elevate NLK levels and impairing Wnt/β-catenin axis	[89]

## Function of circRNAs as biomarkers

The distinct biochemical characteristics of circRNAs has led to the application of circRNAs as potential cancer biomarkers. The circRNAs possess high stability and they have cell-, tissue- and developmental-stage-specific paradigms of expression [90]. Moreover, the circRNAs are demonstrated to be highly conserved among the species and resistant to RNase R activity. The enrichment of circRNAs in exosomes can occur and they are found in body fluids including saliva, plasma and blood. The RNA sequencing has led to the recognition of high number of circRNAs in human peripheral whole blood cell [91]. In high number of cases, the level of circRNAs is higher compared to the corresponding linear RNA isoform [91]. According to this fact, a variety of experiments have emphasized on application of circRNAs as biomarkers in various human cancers [92]. The circ-002059 has a poor expression in gastric tumor compared to the healthy tissues and can influence the distal metastasis and TNM stage, highlighting its function as biomarker in gastric tumor [93]. The circRNA-PVT1 has upregulation in gastric tumor that can affect the overall survival and disease-free survival of patients and through sponging miR-125 family, it functions as tumor-promoting factor [94]. In addition to gastric tumor, the circRNAs have been considered as biomarkers in other kinds of tumors. The circ-0005075 has differential expression between hepatocellular carcinoma and health tissues and its expression can increase tumor size [95]. Moreover, the circ-0001649 has differential expression and shows low expression in hepatocellular carcinoma [96]. Circ-100,876 has high expression in lung cancer and mediates lymph node metastasis and tumor staging, acting as prognostic factor [97].

## CircRNA/EMT axis in cancer metastasis

In this section, we discuss the function of the circRNA/EMT in regulating the invasion of tumor. Based on the role of circRNAs, the interaction of circRNAs and EMT in cancers mediates metastasis. Oncogenic circRNAs induce EMT to increase tumorigenesis, while onco-suppressor circRNAs reduce cancer invasion via EMT inhibition. The field of biology and next-generation sequencing enables the identification of new circRNAs with important functional roles in cancer. The first complexity that arises from circRNAs is that their function in cancer varies and is context-dependent. For instance, circ-0008305 increases the progression of hepatocellular carcinoma via promoting TMED2 level and miR-186 inhibition [43]. Circ-0008305, on the other hand, acts as an oncosuppressor in lung cancer by regulating EMT. Circ-0008305, also known as circPTK2, interacts with miRNAs and EMT regulators such as TGF-β in lung tumor. TGF-β induced EMT in lung tumor promotes tumor cell progression and invasion. Notably, TIF1γ deficiency is required for TGF-β-associated EMT induction in lung tumor [98]. According to these studies, as circRNA induces EMT, it may be capable of suppressing EMT in other cancers.

The expression of certain factors can significantly promote the metastasis, and IGF2BP3 is one of them. USP11 promotes the stability of IGF2BP3 to increase the colorectal cancer metastasis [99]. The upregulation of IGF2BP3 by linc 01305 can lead to overexpression of HTR3A at the mRNA level, which elevates the progression of esophageal tumor [100]. Hence, IGF2BP3 upregulation also increases invasion [101, 102]. IGF2BP3 is the target of circRNAs in regulating the EMT. CircIGHG elevates the malignancy of oral tumor via EMT induction. CircIGHG increases IGF2BP3 level via miR-142-5p inhibition to elevate the progression of oral cancer via an EMT mechanism [103]. Although IGF2BP3 is a target of circRNAs in increasing cancer metastasis, it is not the only mechanism. For instance, the function of circ-0003258 in prostate tumor has been shown to be oncogenic as it induces EMT to elevate cancer metastasis. Circ-0003258 affects two distinct molecular pathways to increase prostate tumor metastasis via EMT. Initially, circ-0003258 sponges miR-635-5p to upregulate ARHGAP5 expression, which then elevates cancer metastasis. Subsequently, circ-0003258 interacts with IGF2BP3 to upregulate HDAC4 and to induce ERK along with EMT [71].

Circ-0001666 impairs breast carcinogenesis via sponging miR-620 to upregulate WNK2 [104]. However, circ-0001666 also enhances the progression of lung and thyroid cancers via regulating ETV4 and AGO1, among others [105, 106]. Circ-0001666 acts as an onco-suppressor to regulate the EMT mechanism in colorectal cancer.

**Fig. 4:**
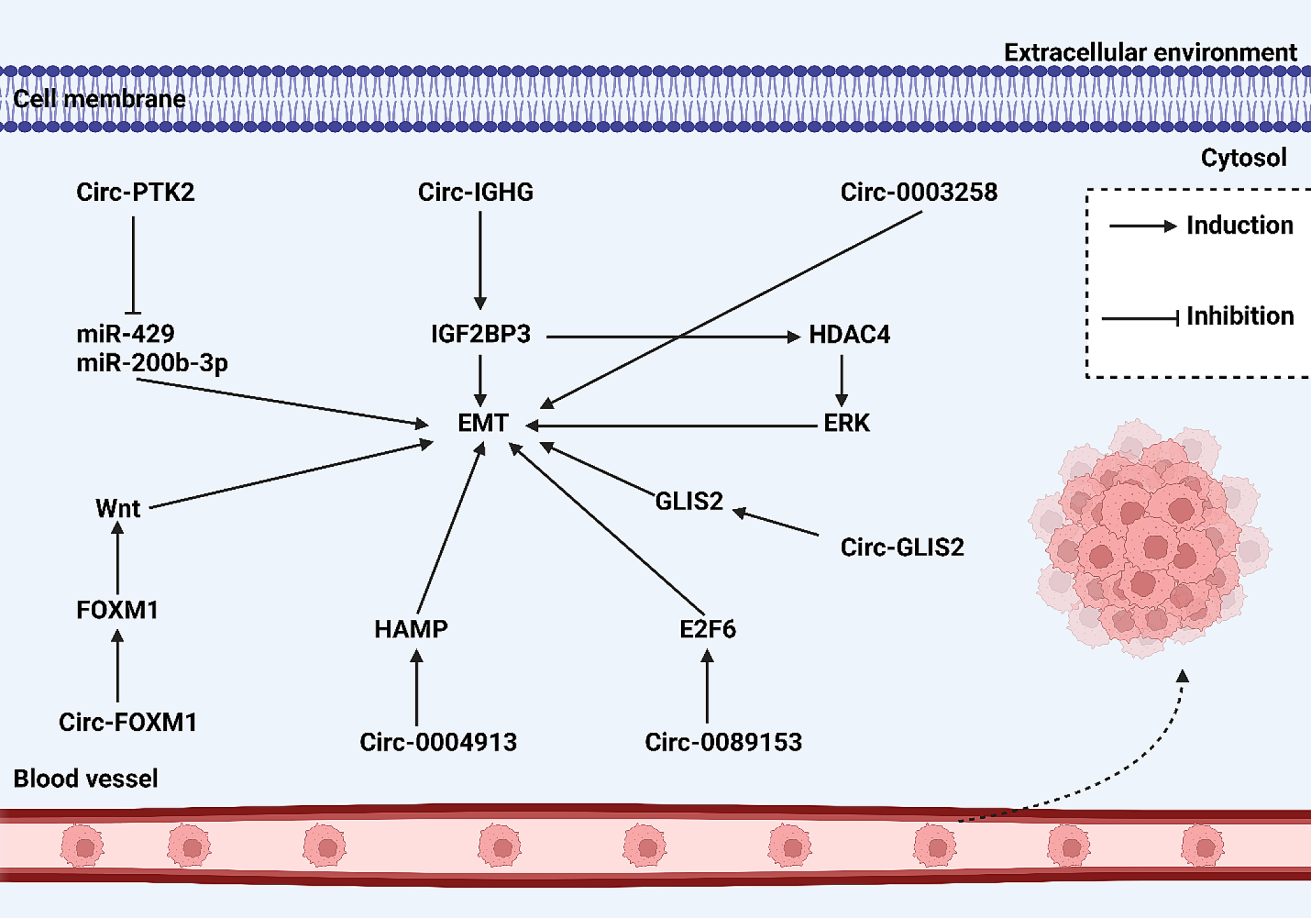
CircRNA/EMT axis in modulating cancer invasion and metastasis. The circRNAs mainly induce EMT in increasing progression of tumor cells. In some cases, including circ-GLIS2, circ-0089153 and circ-0004913, the circRNAs affect the proteins to regulate EMT. However, in other cases, such as circ-PTK2, the circRNAs sponge miRNAs to affect EMT.

“Once miR-576-5p is synthesized in the nucleus, it’s shuttled into the cytoplasm via Exportin5, where it acts to diminish PCDH10 expression, consequently promoting the progression of colorectal cancer. In contrast, the back-splicing event between exon 2 and exon 4 in FAM120B pre-mRNA leads to the creation of circ-0001666. This particular circRNA elevates PCDH10 levels by inhibiting miRNA-576-5p. The outcome is a decrease in the ‘stemness’ of colorectal cancer cells due to the suppression of EMT, which is facilitated by hindering Wnt/β-catenin signaling pathways.” [79]. Circ-0001017 is another example of circRNAs with onco-suppressor function in gliomas, which reduce let-7 g-3p level to upregulate NDST3, suppressing EMT [107]. Drawing from these studies, it becomes clear that the circRNA/EMT axis serves as a key regulatory mechanism in cancer metastasis (Table 3; Fig. 4).

**Table 3:** CircRNA/EMT axis in cancers

CircRNA	Molecular pathway	Cancer type	Remark	Ref
Circ-0082182	miR-411/miR-1205/Wnt	Colorectal cancer	Circ-0082182 stimulates Wnt/β-catenin signaling via miR-411 and miR-1205 sponging in carcinogenesis	[108]
Circ-0001666	miR-1251/SOX4/EMT	Pancreatic cancer	Circ-0001666 upregulates SOX4 via miR-1251 inhibition in EMT acceleration	[86]
Circ-0087429	miR-5003-3p/OGN	Cervical cancer	Circ-0087429 overexpression occurs by EIF4A3 to increase OGN level by miR-5003-3p inhibition in EMT suppression	[78]
Circ-0004913	miR-184/HAMP	Hepatocellular carcinoma	Circ-0004913 increases HAMP level via miR-184 inhibition in suppressing tumor metastasis	[109]
Circ-0089153	miR-2467-3p/E2F6	Breast cancer	Circ-0089153 upregulates E2F6 via miR-2467-3p inhibition in accelerating tumorigenesis	[110]
Circ-GLIS2	GLIS2	Hepatocellular carcinoma	Circ-GLIS2 increases GLIS2 expression in reducing invasion and decreasing levels of EMT-related markers	[111]
Circ-0006732	miR-127-5p/RAB3D	Colorectal cancer	Circ-0006732 increases RAB3D via miR-127-5p inhibition in EMT induction	[112]
Circ-FOXM1	FOXM1/Wnt	Osteosarcoma	Circ-FOXM1 sponges miR-320a and − 320b Upregulation of FOXM1 in Wnt signaling induction Inducing EMT	[113]

## CircRNA/EMT axis in chemoresistance and radioresistance

Chemoresistance is an intriguing concept and can develop in two ways, including intrinsic and acquired chemoresistance. Although understanding drug resistance is a key focus in the past decade, the failure of therapy has not been appropriately solved. Non-coding RNAs are regulators of tumorigenesis, and interestingly, circRNAs can determine therapy response in cancer patients. Hsa-circ-0007883 interacts with FUS to increase the FOXR2 stability in developing paclitaxel resistance in ovarian cancer [114]. Exosomal circ-SFMBT2 upregulates TRIB1 via miR-136-5p sponges to induce docetaxel resistance in prostate cancer [115]. Therefore, the association of circRNAs with downstream targets can change the chemotherapy response in cancer [116].

Paclitaxel is an inhibitor of tumor progression and increases polymerization of microtubules to disturb their stability in inhibiting proliferation. COL5A1 upregulation causes paclitaxel resistance [117]. KHDRBS3 increases MIR17HG level to stimulate glycolysis and paclitaxel insensitivity in ovarian tumor [118]. Upregulation of gp96 and HIF-1α can also result in paclitaxel resistance in cancer [119, 120]. Overexpression of circ-0007534 in endometrial tumor results in paclitaxel resistance. The circ-0007534 elevates ZEB2 level via miR-625 inhibition to accelerate EMT and accelerate paclitaxel insensitivity [121]. Docetaxel functions similarly to paclitaxel and prevents the depolymerization of microtubules to suppress cancer. Upregulation of circ-CRIM1 in nasopharyngeal cancer is a positive indicator of docetaxel resistance in tumor cells. miR-422a decreases FOXQ1 level to inhibit the EMT and mediate docetaxel sensitivity. However, when expression of circ-CRIM1 increases, it sponges miR-422a to upregulate FOXQ1, induce EMT, and increase the docetaxel resistance [122]. The docetaxel potential in prostate tumor therapy increases upon upregulation of circ-Foxo3 and is related to Foxo3 and EMT suppression, thereby reducing the malignancy of cancer cells [123].

**Fig. 5:**
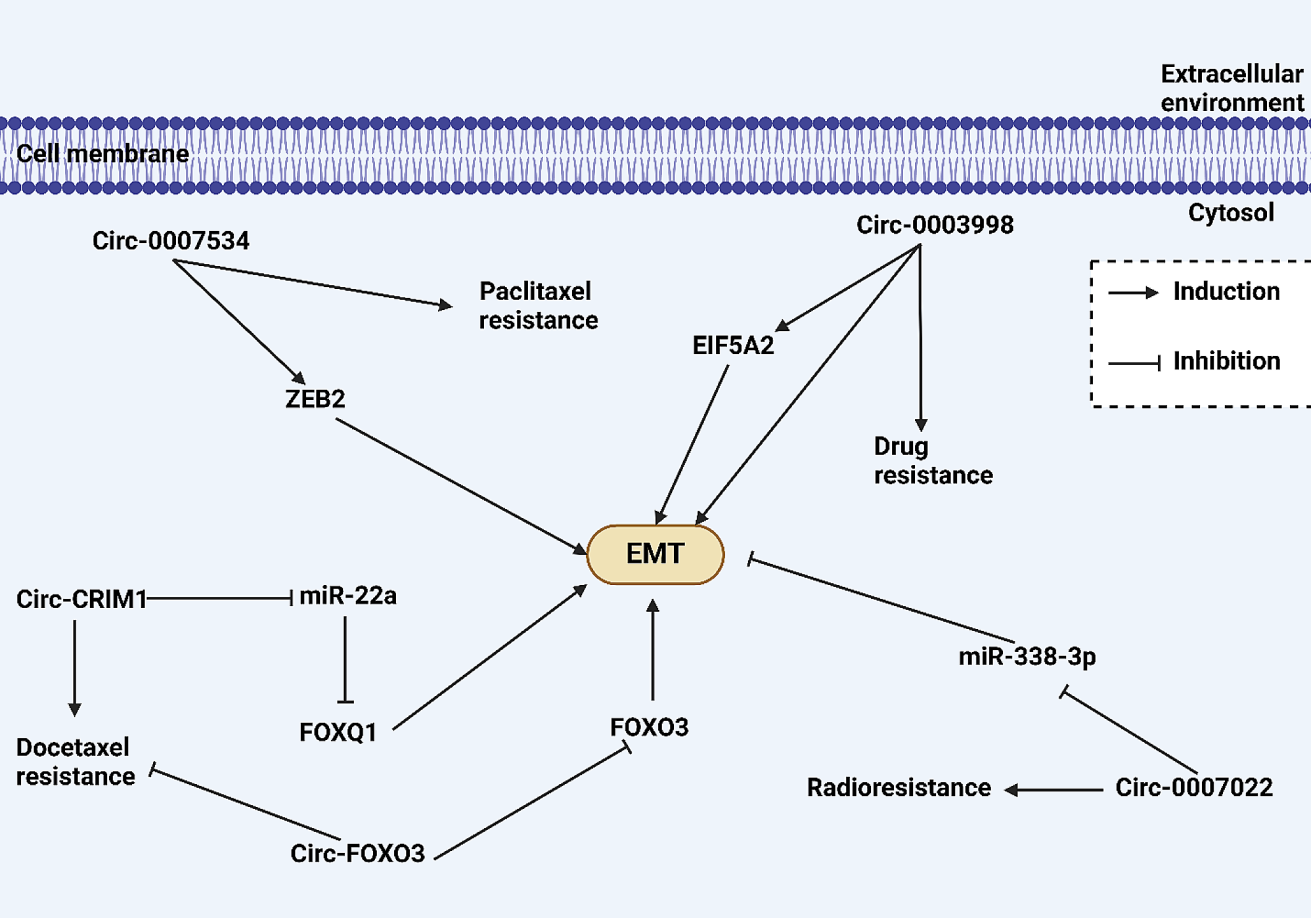
CircRNA/EMT axis in drug resistance and radio-resistance. The upregulation of ZEB2 by circ-0007534 can induce EMT to mediate paclitaxel resistance. Moreover, circ-0003998 increases EIF5A2 levels to mediate EMT-induced drug resistance. The circ-0007022 inhibits miR-338-3p to mediate EMT-induced radioresistance. The sponging of miR-22a by circ-CRIM1 can induce docetaxel resistance

Circ-0003998 is another regulator of tumor progression and can enhance proliferation and invasion in lung tumor via miR-326 inhibition [124]. The function of circ-0003998 in triggering chemoresistance has been evaluated in hepatocellular carcinoma. Circ-0003998 stimulates the EMT to promote tumorigenesis. Additionally, Circ-0003998 reduces miR-218-5p level, which upregulates EIF5A2 [125].Circ-0007022 is present on chromosome 19 and is formed as a result of back-splicing. Circ-0007022 suppresses miR-338-3p to cause radioresistance. Following the inhibition of miR-338-3p by circ-0007022, the levels of NRP1 increased. Two molecular pathways, including PI3K/Akt and EMT, are activated to increase tumor progression and mediate radioresistance [126]. However, only one experiment investigated the function of the circRNA/EMT axis in radioresistance, and more research is required to confirm and expand on the current findings (Fig. 5).

## CircRNA/miRNA/EMT axis

Linear RNA molecules do not encode proteins with a length of less than 24 nucleotides, best known as microRNAs (miRNAs). MicroRNAs sequester mRNA to downregulate gene [127]. The expression level of miRNAs changes during cancer progression and can be used as biomarkers. Furthermore, miRNAs can be sponged by lncRNAs and circRNAs [128, 129]. miRNAs demonstrate dysregulation in tumorigenesis and are biomarkers. The malignancy of cervical tumor depends on the high level of circ-000322, a driver of tumor progression via EMT induction. Upregulation of S100A14 leads to EMT induction and cervical cancer progression. The role of circ-0003221 in enhancing cervical tumorigenesis is related to sponging miR-139-3p to stimulate EMT mechanisms via S100A14 upregulation. Knocking down the expression of circ-0003221 is beneficial for impairing cervical cancer progression via EMT suppression [85]. miR-370 is inhibited by SNHG15 in ovarian cancer to increase the progression of tumor cells [117]. miR-370 overexpression elevates the response of ovarian tumor to cisplatin chemotherapy [130].

**Fig. 6:**
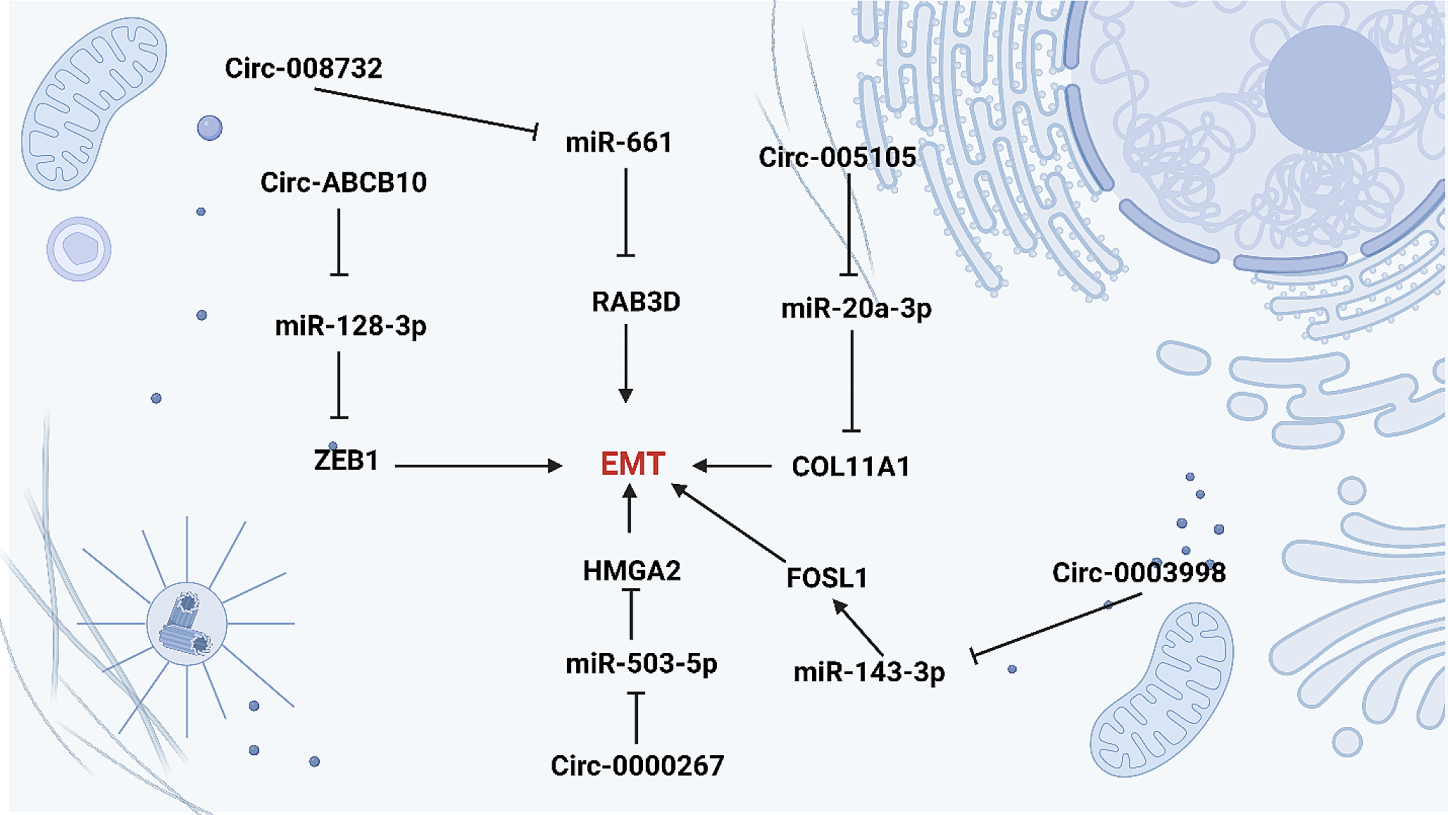
CircRNA/miRNA/EMT axis in human cancers. The circRNAs mainly function as ceRNA to sponge miRNAs in the control of carcinogenesis. The circ-008732 sponges miR-661 to upregulate RAB3D in EMT induction. Moreover, circ-ABCB10 sponges miR-128-3p to stimulate EMT via ZEB1 overexpression. The circ-0000267 suppresses miR-503-5p to increase HMGA2 levels in EMT. Finally, circ-0003998 stimulates EMT through miR-143-3p inhibition

Restoring expression of miR-185-5p is beneficial in suppressing osteosarcoma malignancy, as miR-185-5p reduces CTSE expression [131]. PCAT6 downregulation of miR-185-5p in osteosarcoma can result in TGF-β signaling activation and tumor cell progression [132]. Circ-001569 is capable of sponging miR-185-5p in osteosarcoma, and this interaction results in upregulation of FLOT2, a factor involved in triggering EMT in cancer cells and thus enhancing tumor progression [133]. In esophageal cancer, the interaction of circRNA and miRNA is beneficial in determining the progression. Hsa-circ-0000277 undergoes overexpression in esophageal cancer, and by reducing miR-4766-5p expression, it paves the way for upregulation of LAMA1 to induce EMT [134]. However, not all circRNAs sponge miRNA to induce EMT and tumor metastasis. Sometimes, circRNAs sponge oncogenic miRNAs to suppress EMT mechanisms. Hsa-circ-001988 reduces miR-197-3p level to suppress EMT and the progression of gastric tumor [135]. Moreover, miR-503-5p overexpression can result in EMT in oral cancer as circ-0072387 inhibits miR-503-3p to decrease its expression, disrupt EMT, and minimize tumor progression [136]. More importantly, the interaction of circRNAs and miRNAs can be affected by upstream mediators in cancer. The biogenesis of circ-DOCK5 is inhibited in esophageal cancer by ZEB1. To prevent circ-DOCK5 biogenesis in esophageal cancer, ZEB1 downregulates EIF4E3 and DOCK5 mRNA levels to affect back-splicing between exon 49 and exon 50. When circ-DOXK5 is inhibited, miR-527-3p expression decreases, resulting in decreased TGF-β secretion. TGF-β overexpression then forms a positive feedback loop with ZEB1 to elevate esophageal cancer EMT induction and progression [137]. Table 4; Fig. 6 summarize the function of circRNA/miRNA interactions in the control of EMT mechanisms.

**Table 4:** Modulation of EMT mechanism by circRNA/miRNA axis in tumors

CircRNA	Molecular pathway	Cancer type	Remark	Ref
Circ-0088732	miR-661/RAB3D	Glioma	Circ-0088732 upregulates RAB3D via miR-661 sponging in EMT induction	[138]
Circ-ABCB10	miR-128-3p/ZEB1	Cervical cancer	Circ-ABCB10 upregulates ZEB1 by miR-128-3p inhibition in accelerating tumorigenesis and EMT	[139]
Circ-0000267	miR-503-5p/HMGA2	Gastric cancer	Circ-0000267 upregulates HMGA2 via miR-503-5p suppression in EMT stimulation	[140]
Circ-0000144	miR-217/EMT	Gastric cancer	Circ-0000144 sponges miR-217 in triggering EMT mechanism	[141]
Circ-CORO1C	miR-138-5p/KLF12	Gastric cancer	Circ-CORO1C upregulates KLF12 via miR-138-5p inhibition in EMT	[142]
Circ-03955	miR-3662/MTDH	Osteosarcoma	Circ-03955 promotes MTDH level via miR-3662 suppression in EMT induction	[143]
Circ-006100	miR-195/GPRC5A	Gastric cancer	Circ-006100 upregulates GPRC5A via miR-195 downregulation in EMT	[144]
Circ-0058106	miR-185-3p/Wnt2b/β-catenin/c-Myc	Hypopharyngeal cancer	Circ-0058106 sponges miR-185-3p in inducing Wnt2b/β-catenin/c-Myc in EMT induction	[145]

## CircRNAs regulating EMT-TFs in cancers

### ZEB proteins

The ZEB family of proteins, which includes ZEB1 and ZEB2, is one of the best-known modulator of EMT. Upregulation of ZEB1 in cancer can lead to tumor metastasis. YTHDF3 increases the expression of ZEB1 and promotes its stability at the mRNA level to enhance breast cancer invasion [146]. Induction of PI3K/Akt signaling by BAG4 leads to an increase in cancer metastasis via ZEB1 overexpression [147]. Furthermore, when the expression of ZEB1 increases, it represses E-cadherin to enhance the mobility of tumor cells [148]. Similarly, ZEB2 functions as an oncogenic factor. ZEB2 down-regulation by miR-518a-5p leads to inhibition of breast cancer progression [149]. Paeonol increases the expression level of miR-126-5p to down-regulate ZEB2, thereby decreasing the invasion of lung tumor cells [150]. Therefore, both ZEB1 and ZEB2 proteins are inducers of cancer metastasis, and notably, their expression level is controlled by circRNAs in cancer [151]. Circ-VANGL1 upregulation is in favor of enhancing the metastasis of thyroid cancer cells as it affects ZEB1 expression. Depleting circ-VANGL1 with siRNA is advantageous in reducing the metastasis of tumor cells and decreasing their malignancy. Circ-VANGL1 acts as a ceRNA, decreasing miR-194 expression while increasing ZEB1 expression. As EMT-TFs, ZEB1 stimulates EMT to promote the progression and metastasis of thyroid cancer cells [152]. The expression level of ZEB1 is tightly regulated by miRNAs in cancer. miR-429 is a negative regulator of ZEB1, leading to inhibition of hepatocellular carcinoma cells. In contrast, MAPKAPK5-AS1 increases ZEB1 via adsorption of miR-429 to elevate tumor progression [153]. Moreover, miR-144-3p reduces ZEB1 expression to interfere with lung cancer invasion [154]. Therefore, miRNAs and ZEB1 closely interact to regulate cancer metastasis [155].

However, there are studies demonstrating that circRNAs can suppress ZEB1. Circ-ACAP2 is an inhibitor of EMT in head and neck cancer. Circ-ACAP2 sponges miR-21-5p to suppress STAT3 signaling in cancers, lowering ZEB1 expression and impairing EMT [156]. In addition, ZEB2 is regulated by circRNAs in cancer. Circ-0007534 stimulates EMT in endometrial cancer and, in this way, promotes the progression of tumor cells to mediate paclitaxel resistance. Circ-0007534 increases ZEB2 expression via miR-625 inhibition to trigger the EMT mechanism and paclitaxel resistance [121]. According to these studies, circRNAs regulate ZEB1 and ZEB2, and more research is needed to understand ZEB2 regulation by circRNAs in cancers.

### Slug and Snail

Upregulation of Slug or Snail has been shown to be a driver for the progression and metastasis of cancer cells. Upon overexpression of Snail in cancer, EMT is induced to enhance metastasis. An experiment has shown that upregulation of circ-0023642 can result in a significant enhancement of metastasis in gastric cancer. Circ-0023642 promotes tumor invasion by increasing N-cadherin, vimentin, and Snail expression while decreasing E-cadherin expression [157]. As a result, circRNAs are regulators of snail and slug EMT mechanisms in cancer.

### TGF-β

TGF-β is another important regulator of cancer progression, and abnormal expression can influence cancer metastasis and malignancy. Smad3 is one of the key players of TGF-β signaling. LHPP inhibits phosphorylation of Smad3 to impair the progression and invasion of colorectal cancer [158]. Furthermore, overexpression of TGF-β has been implicated in inhibiting apoptosis in tumor cells and increasing their survival rate [159]. TGF-β and c-Myc signaling pathways are both involved in gastric tumor cell progression and the induction of EMT mechanisms. The expression levels of TGF-β and c-Myc increase in gastric cancer. Notably, circ-CCDC66 stimulates EMT and accelerates the progression of gastric tumors by promoting TGF-β and c-Myc [160].

Circ-VANGL1 interacts with TGF-β to modulate tumorigenesis. miR-150-5p is an inhibitor of TGF-β signaling to prevent EMT mechanism in melanoma. Circ-VANGL1 sponges miR-150-5p to enhance TGF-β expression, resulting in EMT induction and enhancement of melanoma metastasis [161]. Furthermore, TGF-β can regulate expression of circRNAs to affect EMT mechanism. Activation of TGF-β signaling and phosphorylation of Smad2/3 can result in upregulation of circ-Akt1 which in turn increase Akt1 expression via miR-942-5p inhibition to then promote cancer metastasis and EMT induction [162]. Moreover, TGF-β2 promotes the expression level of circ-PRDM5 to enhance COL1A2 expression via miR-92b-3p inhibition, thereby triggering EMT mechanisms and elevating cancer invasion [163].

### Twist

Another important regulator of the EMT mechanism is Twist. Upregulation of Twist can lead to the induction of cancer metastasis via EMT. Silencing EGFR leads to suppression of tamoxifen resistance in breast cancer via reducing Twist expression to impair the EMT mechanism [164]. Twist expression decreases when METTL14 is inhibited, resulting in a reduction in lung cancer progression and invasion [165]. Notably, non-coding RNA molecules can regulate Twist-mediated EMT in cancer [166]. CircRNAs are effective Twist regulators in cancer EMT mechanisms. CircRAB3IP increases the migration and invasion of osteosarcoma cells. miR-580-3p is an inhibitor of Twist1, which reduces the malignancy of osteosarcoma cells. However, circRAB3IP reduces miR-580-3p expression to upregulate Twist1 and promote osteosarcoma malignancy [167]. CircRNA PVT1 is upregulated in cancers and stimulates the Wnt4/β-catenin axis to increase carcinogenesis [168]. Silencing PVT1 is advantageous for reducing the progression of lung cancer and enhancing sensitivity to cisplatin [169]. Reducing the expression level of oncogenic circRNAs is beneficial in impairing tumorigenesis. Upregulation of circ-0001681 results in Twist1 overexpression to enhance thyroid cancer metastasis. Silencing circ-0001681 impairs tumor invasion via EMT inhibition. Mechanistically, circ-0001681 increases Twist1 expression via miR-942-5p sponging to induce EMT and cancer invasion [170]. The interesting point is that the expression level of circRNAs can be increased by Twist during cancer. Circ-10,720 overexpression can lead to EMT induction in hepatocellular carcinoma. Twist1 increases the expression level of circ-10,720 to upregulate vimentin, trigger EMT, and enhance cancer invasion [171]. These studies highlight the fact that EMT-TFs are regulated by circRNAs in cancer, and interconnected molecular pathways need to be highlighted in the near future.

## CircRNA/EMT axis in different cancers

### Brain cancers

One of the malignancies of the central nervous system is glioma, and due to its aggressive nature, the survival rate of patients is 12–14 months. Furthermore, other challenges, including resistance to therapy and recurrence, prevent effective strategies for treatment [172, 173]. Increasing evidence demonstrates the role of circRNAs in regulating the progression of gliomas. Circ-0000215 promotes glioma progression by activating the EMT mechanism, which mediates metastasis. Circ-0000215 reduces the expression level of miR-495-3p to upregulate CXCR2, induce EMT, and pave the way for glioma metastasis [174]. miR-599 is an inhibitor of EMT in esophageal cancer that reduces the progression of tumor cells. Circ-0030018 is an oncogenic factor in esophageal cancer and sponges miR-599 to increase the expression of ENAH. The positive association of circ-0030018 with ENAH expression is vital for inducing EMT and increasing tumor cell invasion and metastasis [175]. Like glioma, EMT induction poses a treatment challenge in glioblastoma. MICAL2 promotes glioblastoma progression by inducing EMT mechanisms via TGF-β upregulation [176]. GRP78 stabilization by UBE2T can lead to EMT induction, thereby increasing the invasion of glioblastoma cells [177]. Anti-cancer agents such as Eriodictyol have been used to reduce ZEB1 expression to suppress EMT in glioblastoma [178]. EMT is commonly induced in glioblastoma. Circ-0001801 stimulates EMT to increase glioblastoma progression. Circ-0001801 enhances the expression level of HMGB3 via miR-628-5p sponging to induce EMT in favor of glioblastoma malignancy [179]. Circ-0067934 also induces EMT in glioblastoma metastasis by inducing PI3K/Akt signaling to mediate EMT and enhance tumor invasion [180].

### Gastrointestinal cancers

- Gastric cancer is a life-threatening disease and is the third leading cause of death [181]. Gastric tumor cells show little response to chemotherapy and radiotherapy and manifest a metastatic nature. The malignancy of gastric cancer cells can be enhanced by circRNAs. An experiment has shown that upregulation of circ-101,882 is beneficial in promoting malignancy and the progression of gastric tumor cells. Circ-101,882 prevents apoptosis and enhances invasion of cancer cells. For this purpose, circ-101,882 promotes vimentin, N-cadherin, and Snail levels while decreasing E-cadherin expression to stimulate EMT [182]. One of the changes in the tumor microenvironment that can increase gastric cancer progression is hypoxia, which promotes CD36 expression to mediate peritoneal invasion [183]. The expression level of non-coding RNAs changes under hypoxia in gastric cancer [184, 185]. Hypoxia induces EMT in gastric cancer and promotes the progression of tumor cells. Circ-0081143 enhances the expression level of EGFR via miR-497-5p inhibition. Since miR-497-5p suppresses EMT, it reduces gastric cancer progression. Conversely, down-regulation of circ-0081143 can lead to EMT induction [186].
- The malignancy of the colon or rectum is known as colorectal cancer, which has high mortality rates [187–189]. The prognosis of colorectal cancer patients is poor despite advances in treatment [190, 191]. miR-338-5p expression increases the progression of colorectal cancer and induces EMT. The expression level of circ-0137008 decreases in colorectal cancer samples, which leads to significant increases in the progression of tumor cells. Circ-0137008 suppresses EMT mechanisms in colorectal cancer via miR-338-5p down-regulation [192]. Silencing circ-0026416 can be considered an impediment to the progression of colorectal cancer. Circ-0026416 increases the progression of colorectal cancer via EMT induction. Circ-0026416 reduces miR-545-3p expression, which subsequently leads to an increased expression level of MYO6 to promote tumor metastasis [193].

### Gynecological cancers

Cervical cancer is a gynecological malignant tumor with high incidence and death rates [194, 195]. The number of cervical cancer cases in developing countries is higher and comprises up to 85% of the global burden [196]. CircRNAs are considered key players in the progression of cervical cancer and the development of therapy resistance [197, 198]. Both proliferation and invasion of cervical tumor cells are enhanced by circ-MYBL2. However, the function of circ-MYBL2 in increasing cervical cancer metastasis is based on regulating EMT mechanisms. Circ-MYBL2 reduces miR-361-3p expression via sponging to enhance lymph node metastasis in cervical tumors [199]. On the other hand, miR-449a overexpression in cervical cancer can impair the progression of tumor cells. Notably, circ-CDK6 is an onco-suppressor factor, and by reducing miR-449a expression, it suppresses EMT in cervical tumors [200]. Due to the aggressive behavior of ovarian cancer cells and the unfavorable prognosis of patients, patients manifest higher death rates compared to cervical and endometrial cancers [201]. Similar to cervical cancer, the function of circRNAs in regulating the progression of ovarian cancer has been well-documented as they affect various molecular pathways [202–204]. A recent experiment has revealed the role of circ-0001756 in regulating ovarian cancer metastasis via affecting EMT mechanisms. Circ-0001756 promotes the expression level of RAB5A by inducing IGF2BP2 expression. Then, RAB5A stimulates the EGFR/MAPK axis to mediate EMT and increasing the progression and metastasis of ovarian tumor cells [205]. Similar to other tumor types, circRNAs regulate miRNA expression by affecting the progression of ovarian cancer. miR-361-5p is an inhibitor of ovarian cancer progression and reduces the expression levels of c-Met to inhibit the Akt/mTOR axis [206]. Circ-0061140 enhances the progression of ovarian cancer and induces EMT mechanisms. Circ-0061140 decreases miR-361-5p expression to promote RAB1A, which in turn triggers EMT-mediated ovarian cancer invasion [207]. Based on these studies, circRNAs are important regulators of EMT in gynecological cancers.

### Urological cancers

The lives of men are commonly threatened by a malignant disease known as prostate cancer, which causes 300,000 deaths annually [188]. Many deaths in prostate cancer patients result from metastasis [208]. Patients with metastasis have an overall survival of 42 months [209], whereas patients with progressive metastatic prostate cancer have a survival of 27 months [210]. New technologies have been developed for the treatment of prostate cancer patients, such as nanoplatforms, but prostate cancer is still an incurable disease. CircRNAs are potential regulators of prostate cancer progression, affecting various molecular pathways [71, 210, 211]. Furthermore, accumulating data emphasizes the role of EMT as an inducer of prostate cancer invasion [212]. The function of circ-0004296 significantly reduces the metastasis of prostate cancer cells. Since EMT is an inducer of prostate cancer invasion, it is regulated by circ-0004296. EIF4A3 interacts with circ-0004296 to suppress nuclear translocation of ETS1, leading to impairment of prostate cancer invasion via EMT suppression [213]. Overexpression of circ-0030586 in prostate cancer can lead to induction of EMT, which enhances the invasion of tumor cells [214]. On the other hand, induction of Akt signaling can result in an increase in the progression of prostate cancer [215, 216]. Circ-0030586 stimulates PI3K/Akt signaling to induce EMT and enhance the invasion and metastasis of prostate tumor cells. This is mediated by E-cadherin downregulation and Twist upregulation [214]. Therefore, the circRNA/EMT axis is a regulator of prostate cancer invasion. The delineation of molecular pathways that can be targeted for treatment of this malignant disease warrants further research [217, 218].

Another malignancy of the genitourinary tract is bladder cancer, with increasing morbidity and mortality around the world [201]. Despite using chemotherapy, radiotherapy, and surgery, the 5-year survival rate of bladder cancer patients is low and has a high economic burden [219, 220]. New therapeutic approaches such as nanotheranostics have been developed for bladder cancer. However, it is highly recommended to focus on the underlying molecular pathways involved in bladder cancer progression. The expression level of circRNAs changes during bladder cancer development and progression. Besides, the metastasis of bladder cancer cells is highly dependent on the activation of the EMT mechanism [221–223]. The interaction of circRNAs with EMT in bladder cancer is important. Circ-BIRC6 is involved in enhancing the progression and metastasis of bladder tumor cells, and its depletion is key in reducing tumor malignancy. Circ-BIRC6 increases the expression level of XBP1 by inhibiting miR-495-3p to enhance the metastasis of bladder cancer cells via EMT induction. As a result, silencing circ-BIRC6 is advantageous in reversing EMT in bladder tumors [224]. In fact, the malignant behavior of bladder cancer cells is mediated via EMT induction. Circ-0006948 contributes to the progression of bladder tumor cells by reducing E-cadherin and increasing N-cadherin, vimentin, and β-catenin levels to stimulate EMT [224]. One of the key inducers of the EMT mechanism in cancer is HMGA2. HMGA2 stimulates Wnt/β-catenin signaling is activated by HMGA2 to mediate EMT and promote the progression of gastric tumor cells [225]. In bladder cancer, upregulation of HMGA2 leads to EMT induction, which enhances the progression of tumor cells. Notably, circ-0000658 promotes the expression level of HMGA2 via miR-498 inhibition to facilitate tumorigenesis in bladder cancer [226]. Therefore, the circRNA/EMT axis is a promising target in bladder cancer therapy (Table 5) [227, 228].

**Table 5:** The role of circRNA/EMT axis in regulating progression of cancers

CircRNA	Molecular pathway	Cancer type	Remark	Ref
Circ-0084904	miR-802/MAL2	Cervical cancer	Circ-0084904 promotes MAL2 expression by miR-802 inhibition in EMT induction	[229]
Circ-UBAP2	miR-361-3p/SOX4	Cervical cancer	Circ-UBAP2 sponges miR-361-3p to promote SOX4 expression in EMT stimulation	[230]
Circ-NFATC3	miR-9-5p/SDC2	Cervical cancer	Circ-NFATC3 promotes SDC2 expression by miR-9-5p inhibition in cancer progression development	[231]
Circ-OXCT1	miR-136/SMAD4	Gastric cancer	Circ-OXCT1 increases SMAD4 expression via miR-136 inhibition in EMT stimulation	[84]
Circ-0049447	miR-324-5p/EMT	Gastric cancer	Circ-0049447 inhibits EMT via miR-324-5p sponging in reducing tumor progression	[232]
Circ-0005230	miR-1299/RHOT1	Gastric cancer	Circ-0005230 increases RHOT1 expression by miR-1299 inhibition in triggering EMT mechanism	[233]
Circ-104,916	-	Gastric cancer	Low expression of circ-104,916 in gastric cancer Circ-104,916 inhibits EMT via E-cadherin upregulation and N-cadherin down-regulation	[234]
Circ-0029803	miR-216b-5p/SKIL	Colorectal cancer	Circ-0029803 increases SKIL expression via miR-216b-5p spongin in EMT induction	[235]
Circ-0053277	miR-2467-3p/MMP-14	Colorectal cancer	Circ-0053277 promotes MMP-14 expression via miR-2467-3p inhibition in increasing cancer progression and EMT induction	[236]
Circ-0000395	miR-432-5p/MYH9	Colorectal cancer	Circ-0000395 enhances MYH9 expression via miR-432-5p inhibition in EMT induction	[237]
Circ-0056618	miR-411-5p/PRRG4	Colorectal cancer	EMT induction by Circ-0056618 via miR-411-5p sponging and upregulating PRRG4 expression	[238]
Circ-0065378	miR-4701-5p/TUSC1	Colorectal cancer	Circ-0065378 reduces miR-4701-5p expression to upregulate TUSC1 expression in impairing EMT	[239]
Circ-0001459	miR-6165/IGF1R	Hepatocellular carcinoma	Circ-0001459 increases IGF1R expression via miR-6165 inhibition in EMT induction	[240]
Circ-0101145	miR-548c-3p/LAMC2	Hepatocellular carcinoma	Circ-0101145 increases LAMC2 expression by miR-548c-3p inhibition in triggering EMT	[241]
Circ-0003528	miR-421/MMP-3	Hepatocellular carcinoma	Circ-0003528 increases MMP-3 expression via miR-421 inhibition in EMT induction	[242]
Circ-PRKAR1B	miR-361-3p/FZD4	Osteosarcoma	EIF4A3 promotes Circ-PRKAR1B expression to sponge miR-361-3p in upregulating FZD4 expression and mediating EMT mechanism	[243]
Circ-ITCH	miR-199a-5p/Klotho	Gastric cancer	Circ-ITCH promotes Klotho expression via miR-199a-5p inhibition in impairing EMT	[235]
Circ-HIPK3	miR-326	Breast cancer	Circ-HIPK3 sponges miR-326 in increasing expression level of EMT markers for cancer progression	[244]

## Conclusion and remarks

Cancer metastasis involves the migration of tumors from their original location to different organs, where they establish new growths. This journey necessitates breaking away from adjacent tissue and entering the circulatory system. Elevated motility and aggressiveness in cancer cells correspond to heightened rates of metastasis. One of the problems of metastasis is related to the diffusion of cancer cells into distant parts of body, challenging eradication of cancer cells. EMT induction is one mechanism facilitating this spread. As detachment of a number of cancer cells from other parts of colony is required for metastasis, the EMT can increase motility of tumor cells to facilitate this process. This paper delves into the influence of EMT-associated pathways in cancer, particularly highlighting the role of epigenetic factors such as circRNAs in modulating EMT. The dysregulation of epigenetic factors in cancer is a common feature and the researchers have focused on understanding the association between epigenetic alterations and EMT in context of cancer progression and EMT. EMT significantly boosts the migratory capabilities of tumor cells, a fact supported by studies across various types of cancers such as those affecting the brain, gastrointestinal tract, reproductive system, urinary system, and blood. It should be noted that function of EMT in cancer is versatile and it is beyond regulation of metastasis and can affect cancer therapy resistance and mediate immunosuppression. Most crucially, circRNAs serve as powerful modulators of EMT pathways in these diverse cancer types. Targeting EMT directly or adjusting the expression levels of circRNAs emerge as two effective approaches for cancer treatment, with the potential to reduce metastatic spread and improve both patient survival rates and prognoses. Since EMT is a reversible process, targeting related circRNAs can significantly change the progression of cancer cells. In exploring the relationship between circRNA and the EMT axis in cancer, two crucial dimensions emerge: metastasis and responsiveness to treatment. On one hand, circRNAs have the ability to either trigger or suppress EMT processes, thus influencing the invasive capabilities of cancer cells. On the other hand, the regulation of EMT by circRNAs plays a key role in determining how cancer cells react to therapeutic interventions. When circRNAs promote EMT, they essentially bolster the malignant properties of tumors, setting the stage for resistance to treatment. Conversely, suppressing EMT through circRNAs can increase a tumor sensitivity to both drug and radiation therapies. Yet, circRNAs are not confined to modulating EMT directly; they can also affect other signaling pathways. A primary function of circRNAs in this context is their ability to sponge miRNAs, thus affecting EMT processes in cancer. Consequently, the circRNA/EMT axis serves as a promising target for therapeutic interventions. EMT-related transcription factors such as ZEB proteins, TGF-β, Twist, and Slug can also be influenced by circRNAs, impacting both the metastasis and severity of cancer. Preliminary studies suggest that circRNAs are pivotal in controlling EMT in cancer, and future research may well center on their potential as biomarkers for treatment outcomes and long-term prognosis in cancer patients. Although significant efforts have been made in understanding the function of circRNAs in regulating EMT in cancers, there are still some limitations to be explored in the future studies. A number of anti-cancer compounds have shown potential in the inhibition of EMT. However, the studies should focus on the compounds regulating circRNA/EMT axis. Moreover, the genetic tools, specially CRISPR/Cas9 system should be introduced for circRNA/EMT axis regulation in human cancer therapy. One of the most promising limitations is lack of emphasizing on nanoparticles for targeting circRNA/EMT axis to impair metastasis and invasion of cancer cells.

The pre-clinical studies provide the valuable insights regarding the metastasis of tumor cells mediated by EMT and then, the function of circRNAs in the modulation of EMT-associated metastasis. Based on the estimates, up to 90% of cancer-related deaths in patients are due to the metastasis. In fact, the metastasis allows the tumor cells to diffuse into various parts of body and develop new colonies that further can proliferate and improve the survival of tumors. Therefore, the modulation of metastasis can be of high importance in clinical level. Since circRNAs can mainly affect metastasis through EMT regulation and their stability is high, they can be utilized as diagnostic and prognostic tools in patients. The changes in the circRNAs in serum or exosomal serum can provide a non-invasive method for the metastasis prediction in patients. In addition to prognostic and diagnostic tool, the circRNAs regulating EMT mechanism can be considered as valuable targets in cancer therapy. However, the drugs have poor ability in the regulation of circRNA regulation and therefore, it is suggested to apply the genetic tools to regulate circRNA expression in cancer therapy.

## References

[1] Babaei G, Aziz SG-G, Jaghi NZZ, EMT (2021) cancer stem cells and autophagy; the three main axes of metastasis. Biomed Pharmacother 133:110909. 10.1016/j.biopha.2020.110909 33227701 10.1016/j.biopha.2020.110909

[2] Talmadge JE, Fidler IJ (2010) J.C.r. AACR centennial series: the biology of cancer metastasis: historical perspective. 70:5649–5669 10.1158/0008-5472.CAN-10-1040 PMC4037932 20610625

[3] Baldawa P, Shirol P, Alur J, Kulkarni VV, JOMFP MP, Metastasis (2017) fro 21:463 10.4103/jomfp.JOMFP_158_17 PMC5763887 29391739

[4] PSJNrc Steeg (2016) Targeting metastasis. 16:201–218 10.1038/nrc.2016.25 PMC7055530 27009393

[5] Nieto MA, Huang RY-J, Jackson RA, Thiery JP (2016) EMT: 2016. Cell 166:21–45. 10.1016/j.cell.2016.06.028 27368099 10.1016/j.cell.2016.06.028

[6] Lo U-G, Lee C-F, Lee M-S, Hsieh (2017) J.-T.J.I.j.o.m.s. The role and mechanism of epithelial-to-mesenchymal transition in prostate cancer progression. 18:2079 10.3390/ijms18102079 PMC5666761 28973968

[7] Moreno-Bueno G, Portillo F, Cano A (2008) Transcriptional regulation of cell polarity in EMT and cancer. Oncogene 27:6958–6969. 10.1038/onc.2008.346 19029937 10.1038/onc.2008.346

[8] Knust E, Bossinger OJS (2002) Composition and formation of intercellular junctions in epithelial cells. 298:1955–1959 10.1126/science.1072161 12471248

[9] Perez-Moreno M, Jamora C, Fuchs EJC (2003) Sticky business: orchestrating cellular signals at adherens junctions. 112:535–548 10.1016/s0092-8674(03)00108-9 12600316

[10] Peinado H, Portillo F, Cano (2004) A.J.I.J.o.D.B. Transcriptional regulation of cadherins during development and carcinogenesis. 48:365–375 10.1387/ijdb.041794hp 15349812

[11] Jeanes A, Gottardi C, Yap AJO (2008) Cadherins and cancer: how does cadherin dysfunction promote tumor progression? 27:6920–6929 10.1038/onc.2008.343 PMC2745643 19029934

[12] Jiang Y, Zhuo X, Wu Y, Fu X, Mao C (2022) PAR2 blockade reverses osimertinib resistance in non-small-cell lung cancer cells via attenuating ERK-mediated EMT and PD-L1 expression. Biochim et Biophys acta Mol cell Res 1869:119144. 10.1016/j.bbamcr.2021.119144 10.1016/j.bbamcr.2021.119144 34599981

[13] Lee DE, Jang EH, Bang C, Kim GL, Yoon SY, Lee DH, Koo J, Na JH, Lee S, Kim JH (2021) Bakuchiol, main component of root bark of Ulmus davidiana var. japonica, inhibits TGF-β-induced in vitro EMT and in vivo metastasis. Arch Biochem Biophys 709:108969. 10.1016/j.abb.2021.108969 34153297 10.1016/j.abb.2021.108969

[14] Lu T, Zheng C, Fan Z (2022) Cardamonin suppressed the migration, invasion, epithelial mesenchymal transition (EMT) and lung metastasis of colorectal cancer cells by down-regulating ADRB2 expression. Pharm Biol 60:1011–1021. 10.1080/13880209.2022.2069823 35645356 10.1080/13880209.2022.2069823 PMC9154753

[15] Bose C, Das U, Kuilya TK, Mondal J, Bhadra J, Banerjee P, Goswami RK, Sinha S (2022) Cananginone abrogates EMT in breast Cancer cells through hedgehog signaling. Chem Biodivers 19:e202100823. 10.1002/cbdv.202100823 35298074 10.1002/cbdv.202100823

[16] Liu J, Chen X, Zhou X, Yi R, Yang Z, Zhao X (2021) Lactobacillus fermentum ZS09 mediates epithelial-mesenchymal transition (EMT) by regulating the transcriptional activity of the Wnt/β-Catenin signalling pathway to inhibit Colon cancer activity. J Inflamm Res 14:7281–7293. 10.2147/jir.S344564 34992417 10.2147/JIR.S344564 PMC8711243

[17] Block CJ, Mitchell AV, Wu L, Glassbrook J, Craig D, Chen W, Dyson G, DeGracia D, Polin L, Ratnam M et al (2021) RNA binding protein RBMS3 is a common EMT effector that modulates triple-negative breast cancer progression via stabilizing PRRX1 mRNA. Oncogene 40:6430–6442. 10.1038/s41388-021-02030-x 34608266 10.1038/s41388-021-02030-x PMC9421946

[18] Kitz J, Lefebvre C, Carlos J, Lowes LE, Allan AL (2021) Reduced Zeb1 expression in prostate Cancer cells leads to an aggressive Partial-EMT phenotype Associated with altered global methylation patterns. Int J Mol Sci 22. 10.3390/ijms222312840 10.3390/ijms222312840 PMC8657557 34884649

[19] Lin XM, Wang ZJ, Lin YX, Chen H (2021) Decreased exosome-delivered mir-486-5p is responsible for the peritoneal metastasis of gastric cancer cells by promoting EMT progress. World J Surg Oncol 19:312. 10.1186/s12957-021-02381-5 34686196 10.1186/s12957-021-02381-5 PMC8539850

[20] Lamouille S, Xu J, Derynck R (2014) Molecular mechanisms of epithelial–mesenchymal transition. Nat Rev Mol Cell Biol 15:178–196. 10.1038/nrm3758 24556840 10.1038/nrm3758 PMC4240281

[21] Xue W, Yang L, Chen C, Ashrafizadeh M, Tian Y, Sun R (2024) Wnt/β-catenin-driven EMT regulation in human cancers. Cell Mol Life Sci 81:79. 10.1007/s00018-023-05099-7 38334836 10.1007/s00018-023-05099-7 PMC10857981

[22] Guo Z, Ashrafizadeh M, Zhang W, Zou R, Sethi G, Zhang X (2023) Molecular profile of metastasis, cell plasticity and EMT in pancreatic cancer: a pre-clinical connection to aggressiveness and drug resistance. Cancer Metastasis Rev. 10.1007/s10555-023-10125-y 37453022 10.1007/s10555-023-10125-y

[23] Zheng Y, Li P, Huang H, Ye X, Chen W, Xu G, Zhang F (2021) Androgen receptor regulates eIF5A2 expression and promotes prostate cancer metastasis via EMT. Cell Death Discovery 7:373. 10.1038/s41420-021-00764-x 34864817 10.1038/s41420-021-00764-x PMC8643356

[24] Zhang X, Luo Y, Cen Y, Qiu X, Li J, Jie M, Yang S, Qin S (2022) MACC1 promotes pancreatic cancer metastasis by interacting with the EMT regulator SNAI1. Cell Death Dis 13:923. 10.1038/s41419-022-05285-8 36333284 10.1038/s41419-022-05285-8 PMC9636131

[25] Xiong T, Huang C, Li J, Yu S, Chen F, Zhang Z, Zhuang C, Li Y, Zhuang C, Huang X et al (2020) LncRNA NRON promotes the proliferation, metastasis and EMT process in bladder cancer. J Cancer 11:1751–1760. 10.7150/jca.37958 32194786 10.7150/jca.37958 PMC7052857

[26] Xiao GY, Tan X, Rodriguez BL, Gibbons DL, Wang S, Wu C, Liu X, Yu J, Vasquez ME, Tran HT et al (2023) EMT activates exocytotic rabs to coordinate invasion and immunosuppression in lung cancer. Proc Natl Acad Sci USA 120:e2220276120. 10.1073/pnas.2220276120 37406091 10.1073/pnas.2220276120 PMC10334751

[27] Liang S, Guo H, Ma K, Li X, Wu D, Wang Y, Wang W, Zhang S, Cui Y, Liu Y et al (2021) A PLCB1-PI3K-AKT Signaling Axis activates EMT to promote Cholangiocarcinoma Progression. Cancer Res 81:5889–5903. 10.1158/0008-5472.can-21-1538 34580062 10.1158/0008-5472.CAN-21-1538 PMC9397629

[28] Papatsirou M, Artemaki PI, Scorilas A, Kontos CK (2020) J.P.M. The role of circular RNAs in therapy resistance of patients with solid tumors. 17:469–490 10.2217/pme-2020-0103 33052780

[29] Hsu M-T, Coca-Prados MJN (1979) Electron microscopic evidence for the circular form of RNA in the cytoplasm of eukaryotic cells. 280:339–340 10.1038/280339a0 460409

[30] Kos A, Dijkema R, Arnberg A, Van der Meide P, Schellekens (1986) H.J.N. The hepatitis delta (δ) virus possesses a circular RNA. 323:558–560 10.1038/323558a0 2429192

[31] Nigro JM, Cho KR, Fearon ER, Kern SE, Ruppert JM, Oliner JD, Kinzler KW, Vogelstein (1991) B J C Scrambled Exons 64:607–613 10.1016/0092-8674(91)90244-s 1991322

[32] Chen X, Han P, Zhou T, Guo X, Song X, Li YJ (2016) S.r. circRNADb: a comprehensive database for human circular RNAs with protein-coding annotations. 6:1–6 10.1038/srep34985 PMC5057092 27725737

[33] Yu J, Yang L, Lu H (2021) The emerging role of circular RNAs in common solid malignant tumors in children. Cancer Cell Int 21:309. 10.1186/s12935-021-01998-6 34116651 10.1186/s12935-021-01998-6 PMC8196486

[34] Jeck WR, Sharpless NE (2014) Detecting and characterizing circular RNAs. Nat Biotechnol 32:453–461. 10.1038/nbt.2890 24811520 10.1038/nbt.2890 PMC4121655

[35] Shen B, Sun K (2021) Exosomal circular RNAs: a new frontier in the metastasis of digestive system tumors. Oncol Lett 22. 10.3892/ol.2021.13087 10.3892/ol.2021.13087 PMC8527826 34691253

[36] Hansen TB, Jensen TI, Clausen BH, Bramsen JB, Finsen B, Damgaard CK, Kjems J (2013) Natural RNA circles function as efficient microRNA sponges. Nature 495:384–388. 10.1038/nature11993 23446346 10.1038/nature11993

[37] Zheng Q, Bao C, Guo W, Li S, Chen J, Chen B, Luo Y, Lyu D, Li Y, Shi G et al (2016) Circular RNA profiling reveals an abundant circHIPK3 that regulates cell growth by sponging multiple miRNAs. Nat Commun 7. 10.1038/ncomms11215 10.1038/ncomms11215 PMC4823868 27050392

[38] Meng L, Chang S, Sang Y, Ding P, Wang L, Nan X, Xu R, Liu F, Gu L, Zheng Y et al (2022) Circular RNA circCCDC85A inhibits breast cancer progression via acting as a miR-550a-5p sponge to enhance MOB1A expression. Breast cancer Research: BCR 24:1. 10.1186/s13058-021-01497-6 34983617 10.1186/s13058-021-01497-6 PMC8725284

[39] Wang R, Wang J, Chen Y, Chen Y, Xi Q, Sun L, Zhang X, Zhang G, Ding X, Shi T et al (2022) Circular RNA circLDLR facilitates cancer progression by altering the miR-30a-3p/SOAT1 axis in colorectal cancer. Cell Death Discovery 8. 10.1038/s41420-022-01110-5 10.1038/s41420-022-01110-5 PMC9276972 35821230

[40] Gao Z, Hu L, Chen F, He C, Hu B, Wang X (2022) Hsa_circular RNA_0001013 exerts oncogenic effects in gastric cancer through the microRNA-136-TWSG1 axis. Am J Translational Res 14:4948–4963 PMC9360872 35958507

[41] Liu J, Dai Z, Li M, Wang B, Zhang X, Li F, Zhang M, Zhang W (2022) Circular RNA circMET contributes to tamoxifen resistance of breast cancer cells by targeting miR-204/AHR signaling. Biochem Biophys Res Commun 627:200–206. 10.1016/j.bbrc.2022.07.097 36049358 10.1016/j.bbrc.2022.07.097

[42] Fu L, Zhang D, Yi N, Cao Y, Wei Y, Wang W, Li L, Circular (2022) RNA circPBX3 promotes cisplatin resistance of ovarian cancer cells via interacting with IGF2BP2 to stabilize ATP7A mRNA expression. Hum Cell 35:1560–1576. 10.1007/s13577-022-00748-8 35907138 10.1007/s13577-022-00748-8

[43] Li H, Luo F, Jiang X, Zhang W, Xiang T, Pan Q, Cai L, Zhao J, Weng D, Li Y et al (2022) CircITGB6 promotes ovarian cancer cisplatin resistance by resetting tumor-associated macrophage polarization toward the M2 phenotype. J Immunother Cancer 10. 10.1136/jitc-2021-004029 10.1136/jitc-2021-004029 PMC8919471 35277458

[44] Chen L, Shan G (2021) CircRNA in cancer: fundamental mechanism and clinical potential. Cancer Lett 505:49–57. 10.1016/j.canlet.2021.02.004 33609610 10.1016/j.canlet.2021.02.004

[45] Chen L, Huang C, Wang X, Shan G (2015) Circular RNAs in eukaryotic cells. Curr Genom 16:312–318 10.2174/1389202916666150707161554 PMC4763969 27047251

[46] Huang G, Li S, Yang N, Zou Y, Zheng D, Xiao T (2017) Recent progress in circular RNAs in human cancers. Cancer Lett 404:8–18 28705773 10.1016/j.canlet.2017.07.002

[47] Zhang Y, Zhang X-O, Chen T, Xiang J-F, Yin Q-F, Xing Y-H, Zhu S, Yang L, Chen (2013) L.-L. circular intronic long noncoding RNAs. Mol Cell 51:792–806 24035497 10.1016/j.molcel.2013.08.017

[48] Li Z, Huang C, Bao C, Chen L, Lin M, Wang X, Zhong G, Yu B, Hu W, Dai L (2015) Exon-intron circular RNAs regulate transcription in the nucleus. Nat Struct Mol Biol 22:256–264 25664725 10.1038/nsmb.2959

[49] Liu X, Wang X, Li J, Hu S, Deng Y, Yin H, Bao X, Zhang QC, Wang G, Wang B (2020) Identification of mecciRNAs and their roles in the mitochondrial entry of proteins. Sci China Life Sci 63:1429–1449 32048164 10.1007/s11427-020-1631-9

[50] Ashwal-Fluss R, Meyer M, Pamudurti NR, Ivanov A, Bartok O, Hanan M, Evantal N, Memczak S, Rajewsky N, Kadener S (2014) circRNA biogenesis competes with pre-mRNA splicing. Mol Cell 56:55–66 25242144 10.1016/j.molcel.2014.08.019

[51] Kristensen LS, Hansen TB, Venø MT, Kjems J (2018) Circular RNAs in cancer: opportunities and challenges in the field. Oncogene 37:555–565 28991235 10.1038/onc.2017.361 PMC5799710

[52] Xue C, Li G, Zheng Q, Gu X, Bao Z, Lu J, Li L (2022) The functional roles of the circRNA/Wnt axis in cancer. Mol Cancer 21. 10.1186/s12943-022-01582-0 10.1186/s12943-022-01582-0 PMC9074313 35513849

[53] Du WW, Zhang C, Yang W, Yong T, Awan FM, Yang BB (2017) Identifying and characterizing circRNA-Protein Interaction. Theranostics 7:4183–4191. 10.7150/thno.21299 29158818 10.7150/thno.21299 PMC5695005

[54] Zang J, Lu D, Xu A (2020) The interaction of circRNAs and RNA binding proteins: an important part of circRNA maintenance and function. J Neurosci Res 98:87–97. 10.1002/jnr.24356 30575990 10.1002/jnr.24356

[55] Okholm TLH, Sathe S, Park SS, Kamstrup AB, Rasmussen AM, Shankar A, Chua ZM, Fristrup N, Nielsen MM, Vang S et al (2020) Transcriptome-wide profiles of circular RNA and RNA-binding protein interactions reveal effects on circular RNA biogenesis and cancer pathway expression. Genome Med 12. 10.1186/s13073-020-00812-8 10.1186/s13073-020-00812-8 PMC7722315 33287884

[56] Wang J, Zhao X, Wang Y, Ren F, Sun D, Yan Y, Kong X, Bu J, Liu M, Xu S (2020) circRNA-002178 act as a ceRNA to promote PDL1/PD1 expression in lung adenocarcinoma. Cell Death Dis 11. 10.1038/s41419-020-2230-9 10.1038/s41419-020-2230-9 PMC6965119 31949130

[57] Li H, Xu JD, Fang XH, Zhu JN, Yang J, Pan R, Yuan SJ, Zeng N, Yang ZZ, Yang H et al (2020) Circular RNA circRNA_000203 aggravates cardiac hypertrophy via suppressing miR-26b-5p and mir-140-3p binding to Gata4. Cardiovascular Res 116:1323–1334. 10.1093/cvr/cvz215 10.1093/cvr/cvz215 PMC7243276 31397837

[58] Hansen TB, Kjems J, Damgaard CK (2013) Circular RNA and miR-7 in cancer. Cancer Res 73:5609–5612. 10.1158/0008-5472.can-13-1568 24014594 10.1158/0008-5472.CAN-13-1568

[59] Chen X, Yang T, Wang W, Xi W, Zhang T, Li Q, Yang A, Wang T (2019) Circular RNAs in immune responses and immune diseases. Theranostics 9:588–607. 10.7150/thno.29678 30809295 10.7150/thno.29678 PMC6376182

[60] Yan L, Chen YG (2020) Circular RNAs in Immune Response and viral infection. Trends Biochem Sci 45:1022–1034. 10.1016/j.tibs.2020.08.006 32900574 10.1016/j.tibs.2020.08.006 PMC7642119

[61] Yang J, Cheng M, Gu B, Wang J, Yan S, Xu D (2020) CircRNA_09505 aggravates inflammation and joint damage in collagen-induced arthritis mice via miR-6089/AKT1/NF-κB axis. Cell Death Dis 11:833. 10.1038/s41419-020-03038-z 33028811 10.1038/s41419-020-03038-z PMC7542153

[62] Wu P, Mo Y, Peng M, Tang T, Zhong Y, Deng X, Xiong F, Guo C, Wu X, Li Y et al (2020) Emerging role of tumor-related functional peptides encoded by lncRNA and circRNA. Mol Cancer 19. 10.1186/s12943-020-1147-3 10.1186/s12943-020-1147-3 PMC6998289 32019587

[63] Wang Y, Liu B, Circular RNA (2020) Diseased Heart Cells 9. 10.3390/cells9051240 10.3390/cells9051240 PMC7290921 32429565

[64] Wang J, Zhu S, Meng N, He Y, Lu R, Yan GR (2019) ncRNA-Encoded peptides or proteins and Cancer. Mol Therapy: J Am Soc Gene Therapy 27:1718–1725. 10.1016/j.ymthe.2019.09.001 10.1016/j.ymthe.2019.09.001 PMC6822234 31526596

[65] Wei Y, Zhang Y, Meng Q, Cui L, Xu C (2019) Hypoxia-induced circular RNA has_circRNA_403658 promotes bladder cancer cell growth through activation of LDHA. Am J Translational Res 11:6838–6849 PMC6895513 31814891

[66] Zhan W, Liao X, Chen Z, Li L, Tian T, Yu L, Wang W, Hu Q (2020) Circular RNA hsa_circRNA_103809 promoted hepatocellular carcinoma development by regulating miR-377-3p/FGFR1/ERK axis. J Cell Physiol 235:1733–1745. 10.1002/jcp.29092 31317555 10.1002/jcp.29092

[67] Miao Z, Li J, Wang Y, Shi M, Gu X, Zhang X, Wei F, Tang X, Zheng L, Xing Y (2023) Hsa_circ_0136666 stimulates gastric cancer progression and tumor immune escape by regulating the miR-375/PRKDC Axis and PD-L1 phosphorylation. Mol Cancer 22. 10.1186/s12943-023-01883-y 10.1186/s12943-023-01883-y PMC10718020 38093288

[68] Zhang C, Zhou X, Geng X, Zhang Y, Wang J, Wang Y, Jing J, Zhou X, Pan W (2021) Circular RNA hsa_circ_0006401 promotes proliferation and metastasis in colorectal carcinoma. Cell Death Dis 12:443. 10.1038/s41419-021-03714-8 33947841 10.1038/s41419-021-03714-8 PMC8097074

[69] Liu Y, Zhang H, Zhang W, Xiang L, Yin Z, Xu H, Lu P, Ma Y, Xiong L, Zhang X et al (2022) circ_0004140 promotes LUAD tumor progression and immune resistance through circ_0004140/miR-1184/CCL22 axis. Cell Death Discovery 8:181. 10.1038/s41420-022-00983-w 35396377 10.1038/s41420-022-00983-w PMC8993797

[70] Shen X, Kong S, Ma S, Shen L, Zheng M, Qin S, Qi J, Wang Q, Cui X, Ju S (2022) Hsa_circ_0000437 promotes pathogenesis of gastric cancer and lymph node metastasis. Oncogene 41:4724–4735. 10.1038/s41388-022-02449-w 36109630 10.1038/s41388-022-02449-w

[71] Yu YZ, Lv DJ, Wang C, Song XL, Xie T, Wang T, Li ZM, Guo JD, Fu DJ, Li KJ et al (2022) Hsa_circ_0003258 promotes prostate cancer metastasis by complexing with IGF2BP3 and sponging miR-653-5p. Mol Cancer 21. 10.1186/s12943-021-01480-x 10.1186/s12943-021-01480-x PMC8729084 34986849

[72] Zhong J, Xu A, Xu P, Su M, Wang P, Liu Z, Li B, Liu C, Jiang N (2023) Circ_0000235 targets MCT4 to promote glycolysis and progression of bladder cancer by sponging miR-330-5p. Cell Death Discovery 9:283. 10.1038/s41420-023-01582-z 37532687 10.1038/s41420-023-01582-z PMC10397263

[73] Ju C, Zhou M, Du D, Wang C, Yao J, Li H, Luo Y, He F, He J (2023) EIF4A3-mediated circ_0042881 activates the RAS pathway via miR-217/SOS1 axis to facilitate breast cancer progression. Cell Death Dis 14. 10.1038/s41419-023-06085-4 10.1038/s41419-023-06085-4 PMC10457341 37626035

[74] Zeng Y, Du W, Huang Z, Wu S, Ou X, Zhang J, Peng C, Sun X, Tang H (2023) Hsa_circ_0060467 promotes breast cancer liver metastasis by complexing with eIF4A3 and sponging miR-1205. Cell Death Discovery 9. 10.1038/s41420-023-01448-4 10.1038/s41420-023-01448-4 PMC10169853 37160894

[75] Li H, Jiao W, Song J, Wang J, Chen G, Li D, Wang X, Bao B, Du X, Cheng Y et al (2023) circ-hnRNPU inhibits NONO-mediated c-Myc transactivation and mRNA stabilization essential for glycosylation and cancer progression. J Experimental Clin cancer Research: CR 42. 10.1186/s13046-023-02898-5 10.1186/s13046-023-02898-5 PMC10666356 37993881

[76] Zhang Y, Wang K, Yang D, Liu F, Xu X, Feng Y, Wang Y, Zhu S, Gu C, Sheng J et al (2023) Hsa_circ_0094606 promotes malignant progression of prostate cancer by inducing M2 polarization of macrophages through PRMT1-mediated arginine methylation of ILF3. Carcinogenesis 44:15–28. 10.1093/carcin/bgac091 36394342 10.1093/carcin/bgac091

[77] Lv J, Zhang Y, Yang M, Qiao L, Wang H, Jiang H, Fu M, Qin J, Xu S (2024) Hsa_circ_0013561 promotes epithelial-mesenchymal transition and tumor progression by regulating ANXA2 via miR-23b-3p in ovarian cancer. Cancer Gene Ther 31:108–118. 10.1038/s41417-023-00686-z 38102461 10.1038/s41417-023-00686-z PMC10794140

[78] Yang M, Hu H, Wu S, Ding J, Yin B, Huang B, Li F, Guo X, Han L (2022) EIF4A3-regulated circ_0087429 can reverse EMT and inhibit the progression of cervical cancer via mir-5003-3p-dependent upregulation of OGN expression. J Experimental Clin cancer Research: CR 41. 10.1186/s13046-022-02368-4 10.1186/s13046-022-02368-4 PMC9069757 35513835

[79] Zhou J, Wang L, Sun Q, Chen R, Zhang C, Yang P, Tan Y, Peng C, Wang T, Jin C et al (2021) Hsa_circ_0001666 suppresses the progression of colorectal cancer through the miR-576-5p/PCDH10 axis. Clin Translational Med 11:e565. 10.1002/ctm2.565 10.1002/ctm2.565 PMC8567033 34841662

[80] Yang T, Sun J, Wang W, Li D, Yang X, Jia A, Ma Y, Fan Z (2022) Hsa_circ_0006732 regulates colorectal cancer cell proliferation, invasion and EMT by miR-127-5p/RAB3D axis. Mol Cell Biochem 477:2751–2760. 10.1007/s11010-022-04458-5 35616807 10.1007/s11010-022-04458-5

[81] Geng CH, Zhang XS, He M, Gao P, Zhao HW (2022) Circ_0000799 promotes proliferation and invasion in colorectal cancer and epithelial-mesenchymal transition. J Gastrointest Oncol 13:3090–3099. 10.21037/jgo-22-1176 36636042 10.21037/jgo-22-1176 PMC9830346

[82] Luo N, Sulaiman Z, Wang C, Ding J, Chen Y, Liu B, Cheng Z, Liu S (2022) Hsa_circ_0000497 and hsa_circ_0000918 contributed to peritoneal metastasis of ovarian cancer via ascites. J Translational Med 20:201. 10.1186/s12967-022-03404-9 10.1186/s12967-022-03404-9 PMC9092689 35538537

[83] Ma T, Guo J, Han J, Li L, Ren Y, Huang J, Diao G, Zheng X, Zheng Y (2023) Circ_0001589/miR-1248/HMGB1 axis enhances EMT-mediated metastasis and cisplatin resistance in cervical cancer. Mol Carcinog 62:1645–1658. 10.1002/mc.23605 37431919 10.1002/mc.23605

[84] Liu J, Dai X, Guo X, Cheng A, Mac SM, Wang Z (2020) Circ-OXCT1 suppresses gastric Cancer EMT and metastasis by attenuating TGF-β pathway through the Circ-OXCT1/miR-136/SMAD4 Axis. OncoTargets Therapy 13:3987–3998. 10.2147/ott.s239789 32523351 10.2147/OTT.S239789 PMC7236241

[85] Chi X, Gu X, Chen S, Shen X (2022) Circ_0003221 downregulation restrains cervical Cancer Cell Growth, Metastasis and Angiogenesis by governing the miR-139-3p/S100A14 pathway. Reproductive Sci (Thousand Oaks Calif) 29:1822–1835. 10.1007/s43032-021-00815-9 10.1007/s43032-021-00815-9 35023052

[86] Zhang R, Zhu W, Ma C, Ai K (2021) Silencing of circRNA circ_0001666 represses EMT in pancreatic Cancer through upregulating miR-1251 and downregulating SOX4. Front Mol Biosci 8:684866. 10.3389/fmolb.2021.684866 34055896 10.3389/fmolb.2021.684866 PMC8155604

[87] Li C, Li W, Cao S, Xu J, Qian Y, Pan X, Lei D, Wei D (2021) Circ_0058106 promotes proliferation, metastasis and EMT process by regulating Wnt2b/β-catenin/c-Myc pathway through mir-185-3p in hypopharyngeal squamous cell carcinoma. Cell Death Dis 12. 10.1038/s41419-021-04346-8 10.1038/s41419-021-04346-8 PMC8575998 34750351

[88] Li C, Cai J, Liu W, Gao Z, Li G (2023) Downregulation of circ-STK39 suppresses pancreatic cancer progression by sponging mir-140-3p and regulating TRAM2-mediated epithelial-mesenchymal transition. Apoptosis: Int J Program cell Death 28:1024–1034. 10.1007/s10495-023-01813-9 10.1007/s10495-023-01813-9 37041422

[89] Song J, Liu Q, Han L, Song T, Huang S, Zhang X, He Q, Liang C, Zhu S, Xiong B (2023) Hsa_circ_0009092/miR-665/NLK signaling axis suppresses colorectal cancer progression via recruiting TAMs in the tumor microenvironment. J Experimental Clin cancer Research: CR 42. 10.1186/s13046-023-02887-8 10.1186/s13046-023-02887-8 PMC10680284 38008713

[90] Verduci L, Strano S, Yarden Y, Blandino G (2019) The circ RNA–micro RNA code: emerging implications for cancer diagnosis and treatment. Mol Oncol 13:669–680 30719845 10.1002/1878-0261.12468 PMC6441890

[91] Memczak S, Papavasileiou P, Peters O, Rajewsky N (2015) Identification and characterization of circular RNAs as a New Class of putative biomarkers in human blood. PLoS ONE 10:e0141214. 10.1371/journal.pone.0141214 26485708 10.1371/journal.pone.0141214 PMC4617279

[92] Meng S, Zhou H, Feng Z, Xu Z, Tang Y, Li P, Wu M (2017) CircRNA: functions and properties of a novel potential biomarker for cancer. Mol Cancer 16:94. 10.1186/s12943-017-0663-2 28535767 10.1186/s12943-017-0663-2 PMC5440908

[93] Li P, Chen S, Chen H, Mo X, Li T, Shao Y, Xiao B, Guo J (2015) Using circular RNA as a novel type of biomarker in the screening of gastric cancer. Clin Chim Acta 444:132–136. 10.1016/j.cca.2015.02.018 25689795 10.1016/j.cca.2015.02.018

[94] Chen J, Li Y, Zheng Q, Bao C, He J, Chen B, Lyu D, Zheng B, Xu Y, Long Z et al (2017) Circular RNA profile identifies circPVT1 as a proliferative factor and prognostic marker in gastric cancer. Cancer Lett 388:208–219. 10.1016/j.canlet.2016.12.006 27986464 10.1016/j.canlet.2016.12.006

[95] Shang X, Li G, Liu H, Li T, Liu J, Zhao Q, Wang C (2016) Comprehensive circular RNA profiling reveals that hsa_circ_0005075, a New Circular RNA biomarker, is involved in Hepatocellular Crcinoma Development. Medicine 95:e3811. 10.1097/md.0000000000003811 27258521 10.1097/MD.0000000000003811 PMC4900729

[96] Qin M, Liu G, Huo X, Tao X, Sun X, Ge Z, Yang J, Fan J, Liu L, Qin W (2016) Hsa_circ_0001649: a circular RNA and potential novel biomarker for hepatocellular carcinoma. Cancer Biomark A 16:161–169. 10.3233/cbm-150552 10.3233/CBM-150552 PMC13016540 26600397

[97] Yao JT, Zhao SH, Liu QP, Lv MQ, Zhou DX, Liao ZJ, Nan KJ (2017) Over-expression of CircRNA_100876 in non-small cell lung cancer and its prognostic value. Pathol Res Pract 213:453–456. 10.1016/j.prp.2017.02.011 28343871 10.1016/j.prp.2017.02.011

[98] Wang L, Yang H, Lei Z, Zhao J, Chen Y, Chen P, Li C, Zeng Y, Liu Z, Liu X et al (2016) Repression of TIF1γ by SOX2 promotes TGF-β-induced epithelial–mesenchymal transition in non-small-cell lung cancer. Oncogene 35:867–877. 10.1038/onc.2015.141 25961934 10.1038/onc.2015.141

[99] Huang YY, Zhang CM, Dai YB, Lin JG, Lin N, Huang ZX, Xu TW (2021) USP11 facilitates colorectal cancer proliferation and metastasis by regulating IGF2BP3 stability. Am J Translational Res 13:480–496 PMC7868846 33594305

[100] Huang GW, Chen QQ, Ma CC, Xie LH, Gu J (2021) linc01305 promotes metastasis and proliferation of esophageal squamous cell carcinoma through interacting with IGF2BP2 and IGF2BP3 to stabilize HTR3A mRNA. Int J Biochem Cell Biol 136. 10.1016/j.biocel.2021.106015 10.1016/j.biocel.2021.106015 34022433

[101] Xu Y, Guo Z, Peng H, Guo L, Wang P (2022) IGF2BP3 promotes cell metastasis and is associated with poor patient survival in nasopharyngeal carcinoma. J Cell Mol Med 26:410–421. 10.1111/jcmm.17093 34894048 10.1111/jcmm.17093 PMC8743660

[102] Jiang W, Cheng X, Wang T, Song X, Zheng Y, Wang L (2020) LINC00467 promotes cell proliferation and metastasis by binding with IGF2BP3 to enhance the mRNA stability of TRAF5 in hepatocellular carcinoma. J Gene Med 22:e3134. 10.1002/jgm.3134 31656043 10.1002/jgm.3134

[103] Liu J, Jiang X, Zou A, Mai Z, Huang Z, Sun L, Zhao J (2021) circIGHG-Induced epithelial-to-mesenchymal transition promotes oral squamous cell carcinoma progression via miR-142-5p/IGF2BP3 signaling. Cancer Res 81:344–355. 10.1158/0008-5472.Can-20-0554 33203701 10.1158/0008-5472.CAN-20-0554

[104] Su N, Liu L, He S, Zeng L (2021) Circ_0001666 affects miR-620/WNK2 axis to inhibit breast cancer progression. Genes Genomics 43:947–959. 10.1007/s13258-021-01114-y 34061329 10.1007/s13258-021-01114-y

[105] Wang X, Li R, Feng L, Wang J, Qi Q, Wei W, Yu Z (2022) Hsa_circ_0001666 promotes non-small cell lung cancer migration and invasion through miR-1184/miR-548I/AGO1 axis. Mol Therapy Oncolytics 24:597–611. 10.1016/j.omto.2022.02.011 10.1016/j.omto.2022.02.011 PMC8892028 35284630

[106] Qi Y, He J, Zhang Y, Wang L, Yu Y, Yao B, Tian Z (2021) Circular RNA hsa_circ_0001666 sponges miR–330–5p, miR–193a–5p and miR–326, and promotes papillary thyroid carcinoma progression via upregulation of ETV4. Oncol Rep 45. 10.3892/or.2021.8001 10.3892/or.2021.8001 PMC7934216 33760216

[107] Li T, Li Y, Zhang W, Zhang J (2021) Hsa_circ_0001017 inhibits proliferation and metastasis via regulating the let-7 g-3p/NDST3 axis in glioma. Folia Neuropathol 59:174–188. 10.5114/fn.2021.107115 34284545 10.5114/fn.2021.107115

[108] Liu R, Deng P, Zhang Y, Wang Y, Peng C (2021) Circ_0082182 promotes oncogenesis and metastasis of colorectal cancer in vitro and in vivo by sponging miR-411 and miR-1205 to activate the Wnt/β-catenin pathway. World J Surg Oncol 19. 10.1186/s12957-021-02164-y 10.1186/s12957-021-02164-y PMC7891146 33596920

[109] Wu M, Sun T, Xing L (2020) Circ_0004913 inhibits cell growth, metastasis, and glycolysis by absorbing miR-184 to regulate HAMP in Hepatocellular Carcinoma. Cancer Biother Radiopharm. 10.1089/cbr.2020.3779 33021399 10.1089/cbr.2020.3779

[110] Gao SL, Fan Y, Liu XD, Liu W, Zhao M (2022) circ_0089153 exacerbates breast cancer cells proliferation and metastasis via sponging miR-2467-3p/E2F6. Environ Toxicol 37:1458–1471. 10.1002/tox.23498 35225430 10.1002/tox.23498

[111] Xing W, Zhou PC, Zhang HY, Chen LM, Zhou YM, Cui XF, Liu ZG (2022) Circular RNA circ_GLIS2 suppresses hepatocellular carcinoma growth and metastasis. Liver International: Official J Int Association Study Liver 42:682–695. 10.1111/liv.15097 10.1111/liv.15097 34743403

[112] Yang T, Sun J, Wang W, Li D, Yang X, Jia A, Ma Y, Fan Z (2022) Hsa_circ_0006732 regulates colorectal cancer cell proliferation, invasion and EMT by miR-127-5p/RAB3D axis. Mol Cell Biochem. 10.1007/s11010-022-04458-5 35616807 10.1007/s11010-022-04458-5

[113] Zhang H, Zhou Q, Shen W (2022) Circ-FOXM1 promotes the proliferation, migration and EMT process of osteosarcoma cells through FOXM1-mediated wnt pathway activation. J Orthop Surg Res 17:344. 10.1186/s13018-022-03207-0 35799265 10.1186/s13018-022-03207-0 PMC9261067

[114] Liang YX, Zhang LL, Yang L (2022) circANKRD17(has_circ_0007883) confers paclitaxel resistance of ovarian cancer via interacting with FUS to stabilize FOXR2. Mol Cell Biochem. 10.1007/s11010-022-04548-4 36107285 10.1007/s11010-022-04548-4

[115] Tan X, Song X, Fan B, Li M, Zhang A, Pei L (2022) Exosomal circRNA scm-like with four malignant brain tumor domains 2 (circ-SFMBT2) enhances the docetaxel resistance of prostate cancer via the microRNA-136-5p/tribbles homolog 1 pathway. Anticancer Drugs 33:871–882. 10.1097/cad.0000000000001365 36136987 10.1097/CAD.0000000000001365

[116] Hashemi M, Ghadyani F, Hasani S, Olyaee Y, Raei B, Khodadadi M, Ziyarani MF, Basti FA, Tavakolpournegari A, Matinahmadi AJJoDDS; et al. (2022) Nanoliposomes for doxorubicin delivery: reversing drug resistance, stimuli-responsive carriers and clinical translation. 104112

[117] Zhang J, Zhang J, Wang F, Xu X, Li X, Guan W, Men T, Xu G (2021) Overexpressed COL5A1 is correlated with tumor progression, paclitaxel resistance, and tumor-infiltrating immune cells in ovarian cancer. J Cell Physiol 236:6907–6919. 10.1002/jcp.30350 33655494 10.1002/jcp.30350

[118] Wu X, Qiu L, Feng H, Zhang H, Yu H, Du Y, Wu H, Zhu S, Ruan Y, Jiang H (2022) KHDRBS3 promotes paclitaxel resistance and induces glycolysis through modulated MIR17HG/CLDN6 signaling in epithelial ovarian cancer. Life Sci 293. 10.1016/j.lfs.2022.120328 10.1016/j.lfs.2022.120328 35051418

[119] Mirzaei S, Zarrabi A, Asnaf SE, Hashemi F, Zabolian A, Hushmandi K, Raei M, Goharrizi MASB, Makvandi P, Samarghandian SJ (2021) L.s. The role of microRNA-338-3p in cancer: growth, invasion, chemoresistance, and mediators. 268:119005 10.1016/j.lfs.2020.119005 33421526

[120] Tian T, Han J, Huang J, Li S, Pang H (2021) Hypoxia-Induced Intracellular and Extracellular Heat shock protein gp96 increases Paclitaxel-Resistance and facilitates Immune Evasion in breast Cancer. Front Oncol 11:784777. 10.3389/fonc.2021.784777 34988020 10.3389/fonc.2021.784777 PMC8722103

[121] Yi H, Han Y, Li S (2022) Oncogenic circular RNA circ_0007534 contributes to paclitaxel resistance in endometrial cancer by sponging miR-625 and promoting ZEB2 expression. Front Oncol 12:985470. 10.3389/fonc.2022.985470 35992812 10.3389/fonc.2022.985470 PMC9386306

[122] Hong X, Liu N, Liang Y, He Q, Yang X, Lei Y, Zhang P, Zhao Y, He S, Wang Y et al (2020) Circular RNA CRIM1 functions as a ceRNA to promote nasopharyngeal carcinoma metastasis and docetaxel chemoresistance through upregulating FOXQ1. Mol Cancer 19. 10.1186/s12943-020-01149-x 10.1186/s12943-020-01149-x PMC7023763 32061262

[123] Shen Z, Zhou L, Zhang C, Xu J (2020) Reduction of circular RNA Foxo3 promotes prostate cancer progression and chemoresistance to docetaxel. Cancer Lett 468:88–101. 10.1016/j.canlet.2019.10.006 31593800 10.1016/j.canlet.2019.10.006

[124] Yu W, Jiang H, Zhang H, Li J (2018) Hsa_circ_0003998 promotes cell proliferation and invasion by targeting miR-326 in non-small cell lung cancer. OncoTargets Therapy 11:5569–5577. 10.2147/ott.S174750 30237723 10.2147/OTT.S174750 PMC6135432

[125] Li X, He J, Ren X, Zhao H, Zhao H (2020) Circ_0003998 enhances doxorubicin resistance in hepatocellular carcinoma by regulating miR-218-5p/EIF5A2 pathway. Diagn Pathol 15:141. 10.1186/s13000-020-01056-1 33308276 10.1186/s13000-020-01056-1 PMC7733254

[126] Zhang J, Yu Y, Yin X, Feng L, Li Z, Liu X, Yu X, Li BA (2022) Circ-0007022/miR-338-3p/Neuropilin-1 Axis reduces the radiosensitivity of esophageal squamous cell carcinoma by activating Epithelial-To-Mesenchymal transition and PI3K/AKT pathway. Front Genet 13:854097. 10.3389/fgene.2022.854097 35571014 10.3389/fgene.2022.854097 PMC9100939

[127] Chipman LB, Pasquinelli A (2019) E.J.T.i.g. miRNA targeting: growing beyond the seed. *35*, 215–222 10.1016/j.tig.2018.12.005 PMC7083087 30638669

[128] Liu Y, Chen T, Zheng G (2021) Exosome-transmitted circ-CARD6 facilitates posterior capsule opacification development by miR-31/FGF7 axis. Exp Eye Res 207:108572. 10.1016/j.exer.2021.108572 33844960 10.1016/j.exer.2021.108572

[129] Noman MZ, Parpal S, Van Moer K, Xiao M, Yu Y, Arakelian T, Viklund J, De Milito A, Hasmim M, Andersson M (2020) Inhibition of Vps34 reprograms cold into hot inflamed tumors and improves anti–PD-1/PD-L1 immunotherapy. Sci Adv 6:eaax7881 32494661 10.1126/sciadv.aax7881 PMC7190323

[130] Chen XP, Chen YG, Lan JY, Shen ZJ (2014) MicroRNA-370 suppresses proliferation and promotes endometrioid ovarian cancer chemosensitivity to cDDP by negatively regulating ENG. Cancer Lett 353:201–210. 10.1016/j.canlet.2014.07.026 25063739 10.1016/j.canlet.2014.07.026

[131] Wu Y, Zhou W, Yang Z, Li J, Jin Y (2022) Mir-185-5p represses cells growth and metastasis of Osteosarcoma via Targeting cathepsin E. Int J Toxicol 41:115–125. 10.1177/10915818211069270 35213250 10.1177/10915818211069270

[132] Zhu C, Huang L, Xu F, Li P, Li P, Hu F (2020) LncRNA PCAT6 promotes tumor progression in osteosarcoma via activation of TGF-β pathway by sponging miR-185-5p. Biochem Biophys Res Commun 521:463–470. 10.1016/j.bbrc.2019.10.136 31676070 10.1016/j.bbrc.2019.10.136

[133] Xiao B, Zhang X, Li X, Zhao Z (2020) Circ_001569 regulates FLOT2 expression to promote the proliferation, migration, invasion and EMT of osteosarcoma cells through sponging miR-185-5p. Open life Sci 15:476–487. 10.1515/biol-2020-0050 33817236 10.1515/biol-2020-0050 PMC7874578

[134] Zhou PL, Wu Z, Zhang W, Xu M, Ren J, Zhang Q, Sun Z, Han X (2021) Circular RNA hsa_circ_0000277 sequesters mir-4766-5p to upregulate LAMA1 and promote esophageal carcinoma progression. Cell Death Dis 12. 10.1038/s41419-021-03911-5 10.1038/s41419-021-03911-5 PMC8257720 34226522

[135] Sun D, Wang G, Xiao C, Xin Y (2021) Hsa_circ_001988 attenuates GC progression in vitro and in vivo via sponging miR-197-3p. J Cell Physiol 236:612–624. 10.1002/jcp.29888 32592202 10.1002/jcp.29888

[136] Han L, Cheng J, Li A, hsa_circ_0072387 (2021) Suppresses Proliferation, Metastasis, and glycolysis of oral squamous cell carcinoma cells by downregulating miR-503-5p. Cancer Biother Radiopharm 36:84–94. 10.1089/cbr.2019.3371 32302508 10.1089/cbr.2019.3371

[137] Meng L, Zheng Y, Liu S, Ju Y, Ren S, Sang Y, Zhu Y, Gu L, Liu F, Zhao Y et al (2021) ZEB1 represses biogenesis of circ-DOCK5 to facilitate metastasis in esophageal squamous cell carcinoma via a positive feedback loop with TGF-β. Cancer Lett 519:117–129. 10.1016/j.canlet.2021.06.026 34216686 10.1016/j.canlet.2021.06.026

[138] Jin T, Liu M, Liu Y, Li Y, Xu Z, He H, Liu J, Zhang Y, Ke Y (2020) Lcn2-derived circular RNA (hsa_circ_0088732) inhibits cell apoptosis and promotes EMT in Glioma via the miR-661/RAB3D Axis. Front Oncol 10. 10.3389/fonc.2020.00170 10.3389/fonc.2020.00170 PMC7047435 32154171

[139] Feng W, Guo R, Zhang D, Zhang R (2021) Circ-ABCB10 knockdown inhibits the malignant progression of cervical cancer through microRNA-128-3p/ZEB1 axis. Biol Procedures Online 23. 10.1186/s12575-021-00154-8 10.1186/s12575-021-00154-8 PMC8422762 34493213

[140] Cai X, Nie J, Chen L, Yu F (2020) Circ_0000267 promotes gastric cancer progression via sponging MiR-503-5p and regulating HMGA2 expression. Mol Genet Genom Med 8:e1093. 10.1002/mgg3.1093 10.1002/mgg3.1093 PMC7005624 31845519

[141] Ji F, Lang C, Gao P, Sun H (2021) Knockdown of Circ_0000144 suppresses cell proliferation, Migration and Invasion in Gastric Cancer Via sponging MiR-217. J Microbiol Biotechnol 31:784–793. 10.4014/jmb.2102.02005 33958507 10.4014/jmb.2102.02005 PMC9705855

[142] Ma G, Li G, Fan W, Xu Y, Song S, Guo K, Liu Z (2021) Circ-0005105 activates COL11A1 by targeting miR-20a-3p to promote pancreatic ductal adenocarcinoma progression. Cell Death Dis 12:656. 10.1038/s41419-021-03938-8 34183642 10.1038/s41419-021-03938-8 PMC8239051

[143] Wang Z, Deng M, Chen L, Wang W, Liu G, Liu D, Han Z, Zhou Y, Circular RNA (2020) Circ-03955 promotes epithelial-mesenchymal transition in Osteosarcoma by regulating miR-3662/Metadherin Pathway. Front Oncol 10. 10.3389/fonc.2020.545460 10.3389/fonc.2020.545460 PMC7708376 33312941

[144] Liang M, Huang G, Liu Z, Wang Q, Yu Z, Liu Z, Lin H, Li M, Zhou X, Zheng Y (2019) Elevated levels of hsa_circ_006100 in gastric cancer promote cell growth and metastasis via miR-195/GPRC5A signalling. Cell Prolif 52:e12661. 10.1111/cpr.12661 31318114 10.1111/cpr.12661 PMC6797516

[145] Fan Y, Liu M, Liu A, Cui N, Chen Z, Yang Q, Su A (2021) Depletion of circular RNA circ_CORO1C suppresses gastric Cancer Development by modulating miR-138-5p/KLF12 Axis. Cancer Manage Res 13:3789–3801. 10.2147/cmar.S290629 10.2147/CMAR.S290629 PMC8123969 34007212

[146] Lin Y, Jin X, Nie Q, Chen M, Guo W, Chen L, Li Y, Chen X, Zhang W, Chen H et al (2022) YTHDF3 facilitates triple-negative breast cancer progression and metastasis by stabilizing ZEB1 mRNA in an m(6)A-dependent manner. Annals Translational Med 10:83. 10.21037/atm-21-6857 10.21037/atm-21-6857 PMC8848410 35282088

[147] Jiang L, Chen Y, Min G, Wang J, Chen W, Wang H, Wang X, Yao N (2021) Bcl2-associated athanogene 4 promotes the invasion and metastasis of gastric cancer cells by activating the PI3K/AKT/NF-κB/ZEB1 axis. Cancer Lett 520:409–421. 10.1016/j.canlet.2021.08.020 34419501 10.1016/j.canlet.2021.08.020

[148] López-Moncada F, Torres MJ, Lavanderos B, Cerda O, Castellón EA, Contreras HR (2022) SPARC induces E-Cadherin repression and enhances Cell Migration through Integrin αvβ3 and the transcription factor ZEB1 in prostate Cancer cells. Int J Mol Sci 23. 10.3390/ijms23115874 10.3390/ijms23115874 PMC9180154 35682554

[149] Wang L, Guo Z, Zhang S, Zhang X, Sang M, Liu Y, Shan B MIR-518a-5p Targets ZEB2 to Suppress the Migration and Invasion of Breast-cancer Cells. *Alternative therapies in health and medicine* 2022 35986741

[150] Lv J, Zhu S, Chen H, Xu Y, Su Q, Yu G, Ma W (2022) Paeonol inhibits human lung cancer cell viability and metastasis in vitro via miR-126-5p/ZEB2 axis. Drug Dev Res 83:432–446. 10.1002/ddr.21873 34636432 10.1002/ddr.21873

[151] Du Y, Liu X, Zhang S, Chen S, Guan X, Li Q, Chen X, Zhao Y (2021) CircCRIM1 promotes ovarian cancer progression by working as ceRNAs of CRIM1 and targeting miR-383-5p/ZEB2 axis. Reproductive Biology Endocrinology: RB&E 19:176. 10.1186/s12958-021-00857-3 34847936 10.1186/s12958-021-00857-3 PMC8630901

[152] Xiang Y, Wang W, Gu J, Shang J, Circular (2022) RNA VANGL1 Facilitates Migration and Invasion of Papillary Thyroid Cancer by Modulating the miR-194/ZEB1/EMT Axis. *Journal of oncology 2022*, 4818651, 10.1155/2022/4818651 10.1155/2022/4818651 PMC8923752 35300347

[153] Peng Z, Ouyang X, Wang Y, Fan Q (2022) MAPKAPK5-AS1 drives the progression of hepatocellular carcinoma via regulating miR-429/ZEB1 axis. BMC Mol cell Biology 23. 10.1186/s12860-022-00420-x 10.1186/s12860-022-00420-x PMC9036786 35468721

[154] Liu SW, Yang P, Li FN, Dou RG, Liu JX, Liu GJ (2022) LncRNA B4GALT1-AS1 promotes non-small cell lung cancer cell growth via increasing ZEB1 level by sponging miR-144-3p. Translational cancer Res 11:538–547. 10.21037/tcr-22-296 10.21037/tcr-22-296 PMC8990218 35402178

[155] Wu H, Dong D, Wang J, Yin S, Gong Y, Yang C, Bai Y, Wang J, Du Y (2022) LncRNA NEAT1 promotes the malignant progression of Colorectal Cancer by Targeting ZEB1 via miR-448. Technol Cancer Res Treat 21:15330338221085348. 10.1177/15330338221085348 35695254 10.1177/15330338221085348 PMC9201302

[156] Ma C, Shi T, Qu Z, Zhang A, Wu Z, Zhao H, Zhao H, Chen H (2020) CircRNA_ACAP2 suppresses EMT in Head and Neck squamous cell carcinoma by targeting the miR-21-5p/STAT3 Signaling Axis. Front Oncol 10. 10.3389/fonc.2020.583682 10.3389/fonc.2020.583682 PMC7759648 33363013

[157] Zhou LH, Yang YC, Zhang RY, Wang P, Pang MH, Liang LQ (2018) CircRNA_0023642 promotes migration and invasion of gastric cancer cells by regulating EMT. Eur Rev Med Pharmacol Sci 22:2297–2303. 10.26355/eurrev_201804_14818 29762831 10.26355/eurrev_201804_14818

[158] Hou B, Li W, Xia P, Zhao F, Liu Z, Zeng Q, Wang S, Chang D (2021) LHPP suppresses colorectal cancer cell migration and invasion in vitro and in vivo by inhibiting Smad3 phosphorylation in the TGF-β pathway. Cell Death Discovery 7:273. 10.1038/s41420-021-00657-z 34608127 10.1038/s41420-021-00657-z PMC8490460

[159] Liu F, Shi Z, Bao W, Zheng J, Chen K, Lin Z, Song HN, Luo X, Dong Q, Jiang L et al (2022) ZIC2 promotes colorectal cancer growth and metastasis through the TGF-β signaling pathway. Exp Cell Res 415:113118. 10.1016/j.yexcr.2022.113118 35390314 10.1016/j.yexcr.2022.113118

[160] Xu G, Chen Y, Fu M, Zang X, Cang M, Niu Y, Zhang W, Zhang Y, Mao Z, Shao M et al (2020) Circular RNA CCDC66 promotes gastric cancer progression by regulating c-Myc and TGF-β signaling pathways. J Cancer 11:2759–2768. 10.7150/jca.37718 32226494 10.7150/jca.37718 PMC7086271

[161] Zhou H, Wu J, Leng S, Hou C, Mo L, Xie X, Wang L, Xu Y (2021) Knockdown of circular RNA VANGL1 inhibits TGF-β-induced epithelial-mesenchymal transition in melanoma cells by sponging miR-150-5p. J Cell Mol Med 25:10837–10845. 10.1111/jcmm.16887 34750955 10.1111/jcmm.16887 PMC8642688

[162] Ou R, Mo L, Tang H, Leng S, Zhu H, Zhao L, Ren Y, Xu Y (2020) circRNA-AKT1 sequesters mir-942-5p to Upregulate AKT1 and promote cervical Cancer progression. Mol Therapy Nucleic Acids 20:308–322. 10.1016/j.omtn.2020.01.003 10.1016/j.omtn.2020.01.003 PMC7078494 32193155

[163] Huang P, Hu Y, Duan Y (2022) TGF-β2-induced circ-PRDM5 regulates migration, invasion, and EMT through the miR-92b-3p/COL1A2 pathway in human lens epithelial cells. J Mol Histol 53:309–320. 10.1007/s10735-021-10053-7 35083632 10.1007/s10735-021-10053-7

[164] Takeda T, Tsubaki M, Matsuda T, Kimura A, Jinushi M, Obana T, Takegami M, Nishida S (2022) EGFR inhibition reverses epithelial–mesenchymal transition, and decreases tamoxifen resistance via snail and twist downregulation in breast cancer cells. Oncol Rep 47. 10.3892/or.2022.8320 10.3892/or.2022.8320 35445730

[165] Yang F, Yuan WQ, Li J, Luo YQ (2021) Knockdown of METTL14 suppresses the malignant progression of non-small cell lung cancer by reducing twist expression. Oncol Lett 22:847. 10.3892/ol.2021.13108 34733365 10.3892/ol.2021.13108 PMC8561617

[166] Tzeng HE, Tang CH, Tsai CH, Chiu CH, Wu MH, Yen Y (2021) ET-1 promotes epithelial-mesenchymal transition in oral squamous cell carcinoma cells via the microRNA-489-3p /TWIST Axis. OncoTargets Therapy 14:5005–5018. 10.2147/ott.S294312 34675545 10.2147/OTT.S294312 PMC8502871

[167] Tang G, Liu L, Xiao Z, Wen S, Chen L, Yang P (2021) CircRAB3IP upregulates twist family BHLH transcription factor (TWIST1) to promote osteosarcoma progression by sponging miR-580-3p. Bioengineered 12:3385–3397. 10.1080/21655979.2021.1948487 34224315 10.1080/21655979.2021.1948487 PMC8806556

[168] Yu F, Lin Y, Ai MM, Tan GJ, Huang JL, Zou ZR (2021) Knockdown of circular RNA hsa_circ_PVT1 inhibited Laryngeal Cancer Progression via preventing wnt4/β-Catenin signaling pathway activation. Front cell Dev Biology 9. 10.3389/fcell.2021.658115 10.3389/fcell.2021.658115 PMC8322683 34336825

[169] Cao L, Zhou X, Ding X, Gao D (2021) Knockdown of circ–PVT1 inhibits the progression of lung adenocarcinoma and enhances the sensitivity to cisplatin via the miR–429/FOXK1 signaling axis. Mol Med Rep 24. 10.3892/mmr.2021.12323 10.3892/mmr.2021.12323 PMC8365593 34328193

[170] Wang T, Huang Y (2021) Downregulation of hsa_circ_0001681 suppresses epithelial-mesenchymal transition in thyroid carcinoma via targeting to miR-942-5p/TWIST1 signaling pathway. J Bioenerg Biomembr 53:609–620. 10.1007/s10863-021-09907-2 34164767 10.1007/s10863-021-09907-2

[171] Meng J, Chen S, Han JX, Qian B, Wang XR, Zhong WL, Qin Y, Zhang H, Gao WF, Lei YY et al (2018) Twist1 regulates Vimentin through Cul2 circular RNA to promote EMT in Hepatocellular Carcinoma. Cancer Res 78:4150–4162. 10.1158/0008-5472.Can-17-3009 29844124 10.1158/0008-5472.CAN-17-3009

[172] Frosina G (2013) Development of therapeutics for high grade gliomas using orthotopic rodent models. Curr Med Chem 20:3272–3299. 10.2174/0929867311320260011 23521683 10.2174/0929867311320260011

[173] Hansen TB, Wiklund ED, Bramsen JB, Villadsen SB, Statham AL, Clark SJ, Kjems J (2011) miRNA-dependent gene silencing involving Ago2-mediated cleavage of a circular antisense RNA. EMBO J 30:4414–4422. 10.1038/emboj.2011.359 21964070 10.1038/emboj.2011.359 PMC3230379

[174] Mutalifu N, Du P, Zhang J, Akbar H, Yan B, Alimu S, Tong L, Luan X (2020) Circ_0000215 increases the expression of CXCR2 and promoted the Progression of Glioma Cells by sponging miR-495-3p. Technol Cancer Res Treat 19:1533033820957026. 10.1177/1533033820957026 33089764 10.1177/1533033820957026 PMC7586024

[175] Wang C, Tang D, Wang H, Hu G, Hu S, Li L, Min B, Wang Y (2019) Circular RNA hsa_circ_0030018 acts as a sponge of miR-599 to aggravate esophageal carcinoma progression by regulating ENAH expression. J Cell Biochem. 10.1002/jcb.29507 31736156 10.1002/jcb.29507

[176] Pu B, Zhang X, Yan T, Li Y, Liu B, Jian Z, Mahgoub OK, Gu L, Xiong X, Zou N (2021) MICAL2 promotes Proliferation and Migration of Glioblastoma cells through TGF-β/p-Smad2/EMT-Like Signaling Pathway. Front Oncol 11. 10.3389/fonc.2021.735180 10.3389/fonc.2021.735180 PMC8632809 34868922

[177] Huang P, Guo Y, Zhao Z, Ning W, Wang H, Gu C, Zhang M, Qu Y, Zhang H, Song Y (2020) UBE2T promotes glioblastoma invasion and migration via stabilizing GRP78 and regulating EMT. Aging 12:10275–10289. 10.18632/aging.103239 32491994 10.18632/aging.103239 PMC7346020

[178] Lv F, Du Q, Li L, Xi X, Liu Q, Li W, Liu S (2021) Eriodictyol inhibits glioblastoma migration and invasion by reversing EMT via downregulation of the P38 MAPK/GSK-3β/ZEB1 pathway. Eur J Pharmacol 900:174069. 10.1016/j.ejphar.2021.174069 33811837 10.1016/j.ejphar.2021.174069

[179] Chen WL, Jiang L, Wang JS, Liao CX (2019) Circ-0001801 contributes to cell proliferation, migration, invasion and epithelial to mesenchymal transition (EMT) in glioblastoma by regulating miR-628-5p/HMGB3 axis. Eur Rev Med Pharmacol Sci 23:10874–10885. 10.26355/eurrev_201912_19791 31858556 10.26355/eurrev_201912_19791

[180] Xin J, Zhang XY, Sun DK, Tian LQ, Xu P (2019) Up-regulated circular RNA hsa_circ_0067934 contributes to glioblastoma progression through activating PI3K-AKT pathway. Eur Rev Med Pharmacol Sci 23:3447–3454. 10.26355/eurrev_201904_17709 31081099 10.26355/eurrev_201904_17709

[181] Siegel RL, Miller KD, Jemal AJ (2019) C.a.c.j.f.c. Cancer statistics, 2019, *69*, 7–34 10.3322/caac.21551 30620402

[182] Yin GH, Gao FC, Tian J, Zhang WB (2019) Hsa_circ_101882 promotes migration and invasion of gastric cancer cells by regulating EMT. J Clin Lab Anal 33:e23002. 10.1002/jcla.23002 31420917 10.1002/jcla.23002 PMC6868453

[183] Aoki T, Kinoshita J, Munesue S, Hamabe-Horiike T, Yamaguchi T, Nakamura Y, Okamoto K, Moriyama H, Nakamura K, Harada S et al (2022) Hypoxia-Induced CD36 expression in gastric Cancer cells promotes peritoneal metastasis via fatty acid uptake. Ann Surg Oncol. 10.1245/s10434-022-12465-5 36042102 10.1245/s10434-022-12465-5 PMC10085939

[184] Yang H, Hu Y, Weng M, Liu X, Wan P, Hu Y, Ma M, Zhang Y, Xia H, Lv K (2022) Hypoxia inducible lncRNA-CBSLR modulates ferroptosis through m6A-YTHDF2-dependent modulation of CBS in gastric cancer. J Adv Res 37:91–106. 10.1016/j.jare.2021.10.001 35499052 10.1016/j.jare.2021.10.001 PMC9039740

[185] Li H, Cao B, Zhao R, Li T, Xu X, Cui H, Deng H, Gao J, Wei B (2022) circDNMT1 promotes malignant progression of gastric Cancer through Targeting miR-576-3p/Hypoxia Inducible Factor-1 alpha Axis. Front Oncol 12. 10.3389/fonc.2022.817192 10.3389/fonc.2022.817192 PMC9197105 35712504

[186] Tang J, Zhu H, Lin J, Wang H (2021) Knockdown of Circ_0081143 mitigates Hypoxia-Induced Migration, Invasion, and EMT in gastric Cancer cells through the miR-497-5p/EGFR Axis. Cancer Biother Radiopharm 36:333–346. 10.1089/cbr.2019.3512 32678674 10.1089/cbr.2019.3512

[187] Jemal A, Bray F, Center MM, Ferlay J, Ward E, Forman D (2011) Global cancer statistics. Cancer J Clin 61:69–90. 10.3322/caac.20107 10.3322/caac.20107 21296855

[188] Ferlay J, Soerjomataram I, Dikshit R, Eser S, Mathers C, Rebelo M, Parkin DM, Forman D, Bray F (2015) Cancer incidence and mortality worldwide: sources, methods and major patterns in GLOBOCAN 2012. Int J Cancer 136:E359–386. 10.1002/ijc.29210 25220842 10.1002/ijc.29210

[189] Fitzmaurice C, Allen C, Barber RM, Barregard L, Bhutta ZA, Brenner H, Dicker DJ, Chimed-Orchir O, Dandona R, Dandona L et al (2017) Global, Regional, and National Cancer incidence, mortality, years of Life Lost, Years lived with disability, and disability-adjusted life-years for 32 Cancer groups, 1990 to 2015: a systematic analysis for the global burden of Disease Study. JAMA Oncol 3:524–548. 10.1001/jamaoncol.2016.5688 27918777 10.1001/jamaoncol.2016.5688 PMC6103527

[190] Peng HX, Yang L, He BS, Pan YQ, Ying HQ, Sun HL, Lin K, Hu XX, Xu T, Wang S (2017) Combination of preoperative NLR, PLR and CEA could increase the diagnostic efficacy for I-III stage CRC. 31:e22075K.J.J.o.c.l.a 10.1002/jcla.22075 PMC6816914 27686880

[191] Brody HJS (2015) https://doi.org/10./521S1a. Colorectal cancer. Nature 521 10.1038/521S1a 25970450

[192] Yang Z, Zhang J, Lu D, Sun Y, Zhao X, Wang X, Zhou W, He Q, Jiang Z (2020) Hsa_circ_0137008 suppresses the malignant phenotype in colorectal cancer by acting as a microRNA-338-5p sponge. Cancer Cell Int 20. 10.1186/s12935-020-1150-1 10.1186/s12935-020-1150-1 PMC7057602 32158357

[193] Zhang B, Li Y, Wu Q, Xie L, Barwick B, Fu C, Li X, Wu D, Xia S, Chen J et al (2021) Acetylation of KLF5 maintains EMT and tumorigenicity to cause chemoresistant bone metastasis in prostate cancer. Nat Commun 12. 10.1038/s41467-021-21976-w 10.1038/s41467-021-21976-w PMC7969754 33731701

[194] Torre LA, Bray F, Siegel RL, Ferlay J, Lortet-Tieulent J, Jemal A (2015) Global cancer statistics, 2012. Cancer J Clin 65:87–108. 10.3322/caac.21262 10.3322/caac.21262 25651787

[195] Franco EL, Schlecht NF, Saslow D (2003) The epidemiology of cervical cancer. Cancer J (Sudbury Mass) 9:348–359. 10.1097/00130404-200309000-00004 10.1097/00130404-200309000-00004 14690309

[196] Muñoz N (2000) Human papillomavirus and cancer: the epidemiological evidence. J Clin Virology: Official Publication Pan Am Soc Clin Virol 19:1–5. 10.1016/s1386-6532(00)00125-6 10.1016/s1386-6532(00)00125-6 11091143

[197] Jiao J, Zhang T, Jiao X, Huang T, Zhao L, Ma D, Cui B (2020) hsa_circ_0000745 promotes cervical cancer by increasing cell proliferation, migration, and invasion. J Cell Physiol 235:1287–1295. 10.1002/jcp.29045 31256433 10.1002/jcp.29045 PMC6899562

[198] Cen Y, Zhu T, Zhang Y, Zhao L, Zhu J, Wang L, Xu J, Ding T, Xie X, Wang X et al (2022) hsa_circ_0005358 suppresses cervical cancer metastasis by interacting with PTBP1 protein to destabilize CDCP1 mRNA. Mol Therapy Nucleic Acids 27:227–240. 10.1016/j.omtn.2021.11.020 10.1016/j.omtn.2021.11.020 PMC8693350 34976440

[199] Wang J, Li H, Liang Z (2019) circ-MYBL2 serves as a sponge for mir-361-3p promoting Cervical Cancer cells Proliferation and Invasion. OncoTargets Therapy 12:9957–9964. 10.2147/ott.S218976 31819492 10.2147/OTT.S218976 PMC6877451

[200] Zhong P, Guo A, Wang L, Lin X, Feng M, Circular (2022) RNA CDK6 suppresses cervical cancer proliferation and metastasis by sponging miR-449a. Bioengineered 13:4885–4897. 10.1080/21655979.2022.2036898 35152839 10.1080/21655979.2022.2036898 PMC8974052

[201] Siegel RL, Miller KD, Jemal A, Cancer Statistics (2017) *CA: a cancer journal for clinicians* 2017, *67*, 7–30, 10.3322/caac.21387 10.3322/caac.21387 28055103

[202] Hou W, Zhang Y (2021) Circ_0025033 promotes the progression of ovarian cancer by activating the expression of LSM4 via targeting miR-184. Pathol Res Pract 217:153275. 10.1016/j.prp.2020.153275 33285422 10.1016/j.prp.2020.153275

[203] Chen S, Wu W, Li QH, Xie BM, Shen F, Du YP, Zong ZH, Wang LL, Wei XQ, Zhao Y (2021) Circ-NOLC1 promotes epithelial ovarian cancer tumorigenesis and progression by binding ESRP1 and modulating CDK1 and RhoA expression. Cell Death Discovery 7. 10.1038/s41420-020-00381-0 10.1038/s41420-020-00381-0 PMC7822960 33483472

[204] Wang X, Yao Y, Jin M (2020) Circ-0001068 is a novel biomarker for ovarian cancer and inducer of PD1 expression in T cells. Aging 12:19095–19106. 10.18632/aging.103706 33028742 10.18632/aging.103706 PMC7732319

[205] Ji J, Li C, Wang J, Wang L, Huang H, Li Y, Fang J (2022) Hsa_circ_0001756 promotes ovarian cancer progression through regulating IGF2BP2-mediated RAB5A expression and the EGFR/MAPK signaling pathway. Cell Cycle (Georgetown Tex) 21:685–696. 10.1080/15384101.2021.2010166 35113003 10.1080/15384101.2021.2010166 PMC8973336

[206] Ma J, Jing X, Chen Z, Duan Z, Zhang Y (2018) MiR-361-5p decreases the tumorigenicity of epithelial ovarian cancer cells by targeting at RPL22L1 and c-Met signaling. Int J Clin Exp Pathol 11:2588–2596 31938372 PMC6958233

[207] Zhang Y, Di Q, Chen J, Chang M, Ma Y, Yu J (2022) Circ_0061140 contributes to the malignant progression in Ovarian Cancer cells by mediating the RAB1A level through sponging miR-361-5p. Biochem Genet. 10.1007/s10528-022-10200-z 35181843 10.1007/s10528-022-10200-z

[208] Center MM, Jemal A, Lortet-Tieulent J, Ward E, Ferlay J, Brawley O, Bray F (2012) International variation in prostate cancer incidence and mortality rates. Eur Urol 61:1079–1092. 10.1016/j.eururo.2012.02.054 22424666 10.1016/j.eururo.2012.02.054

[209] James ND, Spears MR, Clarke NW, Dearnaley DP, De Bono JS, Gale J, Hetherington J, Hoskin PJ, Jones RJ, Laing R et al (2015) Survival with newly diagnosed metastatic prostate Cancer in the Docetaxel era: data from 917 patients in the control arm of the STAMPEDE trial (MRC PR08, CRUK/06/019). Eur Urol 67:1028–1038. 10.1016/j.eururo.2014.09.032 25301760 10.1016/j.eururo.2014.09.032

[210] Chowdhury S, Bjartell A, Lumen N, Maroto P, Paiss T, Gomez-Veiga F, Birtle A, Kramer G, Kalinka E, Spaëth D et al (2020) Real-world outcomes in First-Line treatment of metastatic castration-resistant prostate Cancer: the prostate Cancer Registry. Target Oncol 15:301–315. 10.1007/s11523-020-00720-2 32500294 10.1007/s11523-020-00720-2 PMC7283204

[211] Kong M, Li H, Yuan W, Mao L, Chen J (2021) The role of Circ_PRKCI/miR-24-3p in the metastasis of prostate cancer. J B U : Official J Balkan Union Oncol 26:949–955 34268958

[212] Ren D, Yang Q, Dai Y, Guo W, Du H, Song L, Peng X (2017) Oncogenic mir-210-3p promotes prostate cancer cell EMT and bone metastasis via NF-κB signaling pathway. Mol Cancer 16:117. 10.1186/s12943-017-0688-6 28693582 10.1186/s12943-017-0688-6 PMC5504657

[213] Mao S, Zhang W, Yang F, Guo Y, Wang H, Wu Y, Wang R, Maskey N, Zheng Z, Li C et al (2021) Hsa_circ_0004296 inhibits metastasis of prostate cancer by interacting with EIF4A3 to prevent nuclear export of ETS1 mRNA. J Experimental Clin cancer Research: CR 40. 10.1186/s13046-021-02138-8 10.1186/s13046-021-02138-8 PMC8543852 34696782

[214] Luo GC, Chen L, Fang J, Yan ZJ (2021) Hsa_circ_0030586 promotes epithelial-mesenchymal transition in prostate cancer via PI3K-AKT signaling. Bioengineered 12:11089–11107. 10.1080/21655979.2021.2008217 34852706 10.1080/21655979.2021.2008217 PMC8810173

[215] Chen H, Zhou L, Wu X, Li R, Wen J, Sha J, Wen X (2016) The PI3K/AKT pathway in the pathogenesis of prostate cancer. Front Bioscience (Landmark Edition) 21:1084–1091. 10.2741/4443 10.2741/4443 27100493

[216] Yi J, Zhu J, Wu J, Thompson CB, Jiang X (2020) Oncogenic activation of PI3K-AKT-mTOR signaling suppresses ferroptosis via SREBP-mediated lipogenesis. Proc Natl Acad Sci USA 117:31189–31197. 10.1073/pnas.2017152117 33229547 10.1073/pnas.2017152117 PMC7733797

[217] Chen SS, Zhang YW, Wang YL, Gao Y, Wang C, Hu Q, Wu TG, Tao SC, Zhao JX, Chen M (2021) [Hsa_circ_0005221 promotes prostate cancer progression through the miR-339-5p/STAT5a pathway]. Zhonghua Nan Ke Xue = Natl J Androl 27:301–308 34914211

[218] Ren X, Cheng J, Zhu M, Chen X, Jiang M, Hu X, Lu Y (2022) Circular RNA circ_0062019 exerts oncogenic properties in prostate cancer via mediating miR-1253/NRBP1 axis. Andrologia 54:e14343. 10.1111/and.14343 34866220 10.1111/and.14343

[219] Antoni S, Ferlay J, Soerjomataram I, Znaor A, Jemal A, Bray F (2017) Bladder Cancer incidence and mortality: A global overview and recent trends. Eur Urol 71:96–108. 10.1016/j.eururo.2016.06.010 27370177 10.1016/j.eururo.2016.06.010

[220] Ploeg M, Aben KK, Kiemeney LA (2009) The present and future burden of urinary bladder cancer in the world. World J Urol 27:289–293. 10.1007/s00345-009-0383-3 19219610 10.1007/s00345-009-0383-3 PMC2694323

[221] Zhang M, Du H, Wang L, Yue Y, Zhang P, Huang Z, Lv W, Ma J, Shao Q, Ma M et al (2020) Thymoquinone suppresses invasion and metastasis in bladder cancer cells by reversing EMT through the Wnt/β-catenin signaling pathway. Chemico-Biol Interact 320. 10.1016/j.cbi.2020.109022 10.1016/j.cbi.2020.109022 32112862

[222] Zhang N, Hua X, Tu H, Li J, Zhang Z, Max C (2021) Isorhapontigenin (ISO) inhibits EMT through FOXO3A/METTL14/VIMENTIN pathway in bladder cancer cells. Cancer Lett 520:400–408. 10.1016/j.canlet.2021.07.041 34332039 10.1016/j.canlet.2021.07.041 PMC9161647

[223] Tan S, Kang Y, Li H, He HQ, Zheng L, Wu SQ, Ai K, Zhang L, Xu R, Zhang XZ et al (2021) circST6GALNAC6 suppresses bladder cancer metastasis by sponging miR-200a-3p to modulate the STMN1/EMT axis. Cell Death Dis 12. 10.1038/s41419-021-03459-4 10.1038/s41419-021-03459-4 PMC7876104 33568625

[224] Zhou L, Wang B, Zhang Y, Yao K, Liu B (2021) Silencing circ–BIRC6 inhibits the proliferation, invasion, migration and epithelial–mesenchymal transition of bladder cancer cells by targeting the miR–495–3p/XBP1 signaling axis. Mol Med Rep 24. 10.3892/mmr.2021.12451 10.3892/mmr.2021.12451 PMC8477182 34542161

[225] Zha L, Zhang J, Tang W, Zhang N, He M, Guo Y, Wang Z (2013) HMGA2 elicits EMT by activating the Wnt/β-catenin pathway in gastric cancer. Dig Dis Sci 58:724–733. 10.1007/s10620-012-2399-6 23135750 10.1007/s10620-012-2399-6

[226] Qiu F, Liu Q, Xia Y, Jin H, Lin Y, Zhao X (2022) Circ_0000658 knockdown inhibits epithelial-mesenchymal transition in bladder cancer via miR-498-induced HMGA2 downregulation. J Experimental Clin cancer Research: CR 41:22. 10.1186/s13046-021-02175-3 10.1186/s13046-021-02175-3 PMC8759287 35031054

[227] Tong L, Yang H, Xiong W, Tang G, Zu X, Qi L (2021) circ_100984-miR-432-3p axis regulated c-Jun/YBX-1/β-catenin feedback loop promotes bladder cancer progression. Cancer Sci 112:1429–1442. 10.1111/cas.14774 33314480 10.1111/cas.14774 PMC8019231

[228] Li G, Guo BY, Wang HD, Lin GT, Lan TJ, Ying H, Xu J (2021) CircRNA hsa_circ_0014130 function as a mir-132-3p sponge for playing oncogenic roles in bladder cancer via upregulating KCNJ12 expression. Cell Biol Toxicol. 10.1007/s10565-021-09668-z 34755307 10.1007/s10565-021-09668-z

[229] Chen L, Li H, Yao D, Zou Q, Yu W, Zhou L (2022) The novel circ_0084904/miR-802/MAL2 axis promotes the development of cervical cancer. Reprod Biol 22:100600. 10.1016/j.repbio.2021.100600 35033901 10.1016/j.repbio.2021.100600

[230] Meng L, Jia X, Yu W, Wang C, Chen J, Liu F, Circular (2020) RNA UBAP2 contributes to tumor growth and metastasis of cervical cancer via modulating miR-361-3p/SOX4 axis. Cancer Cell Int 20:357. 10.1186/s12935-020-01436-z 32760224 10.1186/s12935-020-01436-z PMC7393907

[231] Ma N, Li X, Wei H, Zhang H, Zhang S, Circular RNA (2021) circNFATC3 acts as a mir-9-5p sponge to promote cervical cancer development by upregulating SDC2. Cell Oncol (Dordrecht) 44:93–107. 10.1007/s13402-020-00555-z 10.1007/s13402-020-00555-z PMC12980781 32902825

[232] Tang KW, Guo ZX, Wu ZH, Zhou C, Sun J, Wang X, Song YX, Wang ZN (2021) Circ_0049447 acts as a tumor suppressor in gastric cancer through reducing proliferation, migration, invasion, and epithelial-mesenchymal transition. Chin Med J 134:1345–1355. 10.1097/cm9.0000000000001494 33883408 10.1097/CM9.0000000000001494 PMC8183756

[233] Peng YY, Sun D, Xin Y (2022) Hsa_circ_0005230 is up-regulated and promotes gastric cancer cell invasion and migration via regulating the miR-1299/RHOT1 axis. Bioengineered 13:5046–5063. 10.1080/21655979.2022.2036514 35170374 10.1080/21655979.2022.2036514 PMC8973856

[234] Li J, Zhen L, Zhang Y, Zhao L, Liu H, Cai D, Chen H, Yu J, Qi X, Li G (2017) Circ-104916 is downregulated in gastric cancer and suppresses migration and invasion of gastric cancer cells. OncoTargets Therapy 10:3521–3529. 10.2147/ott.S136347 28761361 10.2147/OTT.S136347 PMC5522828

[235] Huang L, Zhu L, Pan S, Xu J, Xie M, Wang W, Xia G (2021) Circ_0029803 serves as the sponge of miR-216b-5p to promote the progression of colorectal cancer by regulating SKIL expression. World J Surg Oncol 19:268. 10.1186/s12957-021-02368-2 34479589 10.1186/s12957-021-02368-2 PMC8417978

[236] Xiao H, Liu M (2020) Circular RNA hsa_circ_0053277 promotes the development of colorectal cancer by upregulating matrix metallopeptidase 14 via mir-2467-3p sequestration. J Cell Physiol 235:2881–2890. 10.1002/jcp.29193 31549406 10.1002/jcp.29193

[237] Fan L, Li W, Jiang H (2022) Circ_0000395 promoted CRC Progression via elevating MYH9 expression by sequestering miR-432-5p. Biochem Genet. 10.1007/s10528-022-10245-0 35759156 10.1007/s10528-022-10245-0

[238] Zhang B, Cao W, Liu Y, Zhao Y, Liu C, Sun B (2022) Circ_0056618 enhances PRRG4 expression by competitively binding to mir-411-5p to promote the malignant progression of colorectal cancer. Mol Cell Biochem. 10.1007/s11010-022-04525-x 35916967 10.1007/s11010-022-04525-x

[239] Yan D, Liu W, Liu Y, Zhu X (2022) Circular RNA circ_0065378 upregulates tumor suppressor candidate 1 by competitively binding with mir-4701-5p to alleviate colorectal cancer progression. J Gastroenterol Hepatol 37:1107–1118. 10.1111/jgh.15862 35434854 10.1111/jgh.15862

[240] Shen D, Zhao H, Zeng P, Ge M, Shrestha S, Zhao W (2022) Circular RNA circ_0001459 accelerates hepatocellular carcinoma progression via the miR-6165/IGF1R axis. Ann N Y Acad Sci 1512:46–60. 10.1111/nyas.14753 35199365 10.1111/nyas.14753 PMC9306989

[241] Jin J, Liu H, Jin M, Li W, Xu H, Wei F (2020) Silencing of hsa_circ_0101145 reverses the epithelial-mesenchymal transition in hepatocellular carcinoma via regulation of the miR-548c-3p/LAMC2 axis. Aging 12:11623–11635. 10.18632/aging.103324 32554866 10.18632/aging.103324 PMC7343517

[242] Zhang B, Zhou J (2022) CircSEC24A (hsa_circ_0003528) interference suppresses epithelial-mesenchymal transition of hepatocellular carcinoma cells via miR-421/MMP3 axis. Bioengineered 13:9049–9062. 10.1080/21655979.2022.2057761 35400271 10.1080/21655979.2022.2057761 PMC9161912

[243] Feng ZH, Zheng L, Yao T, Tao SY, Wei XA, Zheng ZY, Zheng BJ, Zhang XY, Huang B, Liu JH et al (2021) EIF4A3-induced circular RNA PRKAR1B promotes osteosarcoma progression by mir-361-3p-mediated induction of FZD4 expression. Cell Death Dis 12:1025. 10.1038/s41419-021-04339-7 34716310 10.1038/s41419-021-04339-7 PMC8556261

[244] Luo N, Liu S, Li X, Hu Y, Zhang K, Circular RNA (2021) circHIPK3 promotes breast cancer progression via sponging MiR-326. Cell Cycle (Georgetown Tex) 20:1320–1333. 10.1080/15384101.2021.1939476 34152928 10.1080/15384101.2021.1939476 PMC8331001

